# Synthesis and Antitumor Activity of a Series of Novel 1-Oxa-4-azaspiro[4,5]deca-6,9-diene-3,8-dione Derivatives

**DOI:** 10.3390/molecules24050936

**Published:** 2019-03-07

**Authors:** Ze Yang, Qiu Zhong, Shilong Zheng, Guangdi Wang, Ling He

**Affiliations:** 1 Key Laboratory of Drug–Targeting and Drug Delivery System of the Education Ministry, Department of Medicinal Chemistry, West China School of Pharmacy, Sichuan University, Chengdu 610041, Sichuan, China; 2016224055105@stu.scu.edu.cn; 2 Sichuan Engineering Laboratory for Plant–Sourced Drug and Sichuan Research Center for Drug Precision Industrial Technology, Department of Medicinal Chemistry, West China School of Pharmacy, Sichuan University, Chengdu 610041, Sichuan, China; 3 Department of Chemistry, RCMI Cancer Research Center, Xavier University of Louisiana, New Orleans, LA 70125, USA; qzhong@xula.edu (Q.Z.); szheng@xula.edu (S.Z.)

**Keywords:** 1-oxa-4-azaspiro[4.5]deca-6,9-diene-3,8-dione, intramolecular oxidation, antitumor activity

## Abstract

A series of novel 1-oxa-4-azaspiro[4.5]deca-6,9-diene-3,8-diones were designed and synthesized by using 4-aminophenol and α-glycolic acid or lactic acid as starting materials in three or four steps. The key step is the metal-catalyzed oxidative cyclization of the amide to 1-oxa-4-azaspiro[4.5]deca-6,9-diene-3,8-diones (**10a** and **10b**), the reaction conditions of which are investigated and optimized. The anticancer activity of 17 1-oxa-4-azaspiro[4.5]deca-6,9-diene-3,8-dione derivatives was evaluated. Preliminary results showed that 15 compounds have moderate to potent activity against human lung cancer A549, human breast cancer MDA-MB-231, and human cervical cancer HeLa cancer cell lines. Among them, compounds **11b** and **11h** were the most potent against A549 cell line with 0.18 and 0.19 µM of IC_50_, respectively; compounds **11d**, **11h**, and **11k** showed the most potent cytotoxicity against MDA-MB-231 cell line with 0.08, 0.08, and 0.09 µM of IC_50_, respectively, while the activities of **11h**, **11k**, and **12c** against HeLa cell line were the most potent with 0.15, 0.14, and 0.14 µM of IC_50_, respectively. Compound **11h** is a promising candidate for further development, which emerged as the most effective compound overall against the three tested cancer cell lines.

## 1. Introduction

**Figure 1:**
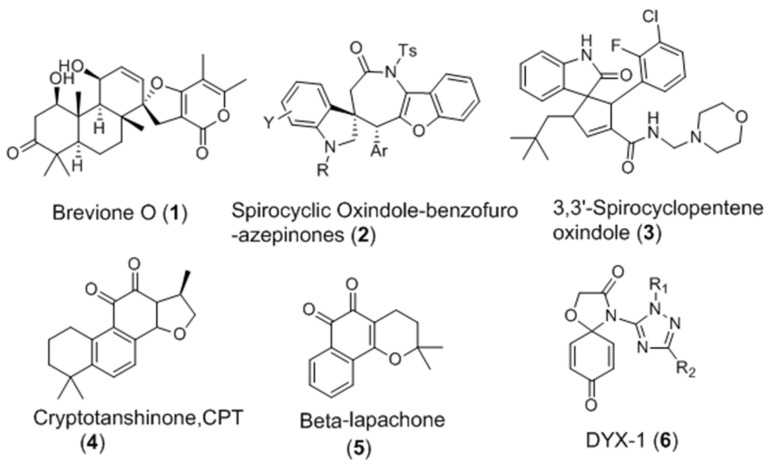
Biologically active spirocyclic and quinone derivatives.

Spirocyclic compounds are an important class of widely distributed natural products [1], such as crotonosine extracted from legumes [2] and brevione O (**1**) extracted from marine fungi [3]. Many spirocycles exhibit different biological activities including antitumor activities. For example, as shown in Figure 1, spirocyclic oxindole-benzofuro-azepinones (**2**) have potent antitumor activity comparable to cisplatin [4] and the IC_50_ value of 3,3′-spirocyclopentene oxindole (**3**) toward wild-type p53-MDM2 was 3.1 nM [5]. The compounds with a quinone scaffold are another class of interesting natural products. They play an important role in the redox process of organisms due to the special character of the quinones, with potential to become attractive cancer chemotherapy drugs such as cryptotanshinone (CPT, **4**), a diterpenoid which exerts antitumor activity through the inhibition of STAT3, [6] and β-lapachone (β-lap, **5**), which is a quinone oxidoreductase 1 (NQO1 or NAD(P)H)-dependent antitumor drug [7,8]. Our previous work hybridized the spirocycle and quinone scaffolds to generate a novel series of 1-oxa-4-azaspiro[4.5]deca-6,9-diene-3,8-diones (**6**) which showed promising anticancer activities [9,10,11,12]. Herein, we report a new synthetic approach to obtain more diversified 1-oxa-4-azaspiro[4.5]deca-6,9-diene-3,8-diones for further anticancer activity assessment.

## 2. Results and Discussion

### 2.1. General Route to Novel 1-Oxa-4-azaspiro[4.5]deca-6,9-diene-3,8-diones

**Scheme 1:**
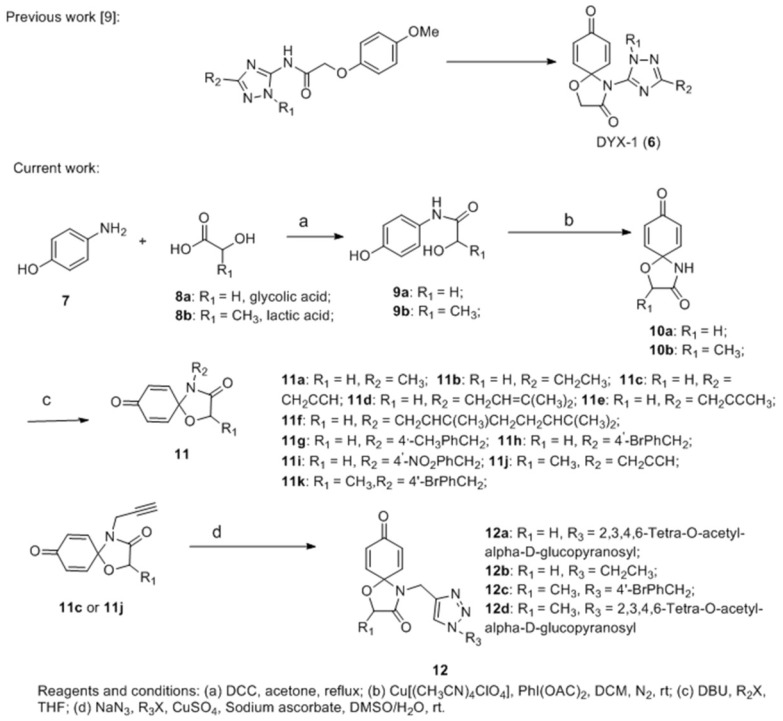
Synthesis of novel 1-oxa-4-azaspiro[4.5]deca-6,9-diene-3,8-diones.

1-Oxa-4-azaspiro[4.5]deca-6,9-diene-3,8-dione derivatives were prepared as shown in Scheme 1. The condensation of 4-hydroxyphenol (**7**) and glycolic acid (**8a**) or lactic acid (**8b**) using *N*,*N*′-dicyclohexylcarbodiimide (DCC) as an activating agent formed 2-hydroxy-*N*- (4-hydroxyphenyl)acetamide (**9a**) or 2-hydroxy-*N*-(4-hydroxyphenyl)propanamide (**9b**). The amides (**9a** and **9b**) were then oxidized by bis(acetoxy)iodobenzene (PhI(OAc)_2_) [13,14,15] to cyclize into 1-oxo-4-azaspiro[4.5]deca-6,9-diene-3,8-dione (**10a**) and 2-methyl-1-oxo-4-azaspiro[4.5] deca-6,9-diene-3,8-dione (**10b**) under the catalysis of tetrakis(acetonitrile)copper (I) perchlorate (Cu[(CH_3_CN)_4_ClO_4_]). The N-alkylation of **10a** and **10b** afforded 1-oxa-4-azaspiro[4.5] deca-6,9-diene-3,8-dione derivatives **11a**–**11k**. Among them, **11c** and **11j** with a terminal alkyne clickly reacted with the azide compounds to afford the triazole derivatives **12a**–**12d** through a one-pot protocol catalyzed by copper (I) generated in situ from copper (II) sulfate and sodium ascorbate [16,17].

### 2.2. Optimization for the Formation of 1-Oxa-4-azaspiro[4.5]deca-6,9-diene-3,8-dione from 2-Hydroxy-N-(4-hydroxyphenyl)acetamide

The metal-catalyzed oxidative cyclization for the conversion from 2-hydroxy-*N*- (4-hydroxyphenyl)acetamide (**9a**) to 1-oxa-4-azaspiro[4.5]deca-6,9-diene-3,8-dione is a new reaction and a key step in the synthesis of the compound. We first investigated and optimized its reagents and conditions. The results in Table 1 show that Cu[(CH_3_CN)_4_ClO_4_] [18] and rhodium acetate (Rh_2_(OAc)_4_) catalyze the reaction to form 1-oxa-4-azaspiro[4.5]deca-6,9-diene-3,8-dione (**10a**) with PhI(OAc)_2_ as an oxidant with yields of 72% and 75%, respectively (Entry 1 and 2), but manganese acetate (Mn(OAc)_2_), ferrous chloride (FeCl_2_), and zinc chloride (ZnCl_2_) failed to catalyze the reactions (Entry 3, 4, and 5). Considering that the metal copper catalyst is more economical and more environmentally friendly, Cu[(CH_3_CN)_4_ClO_4_] was chosen as the reaction catalyst. We chose the oxidant PhI(OAc)_2_ since it displayed slightly better activity than bis(trifluoroacetoxy) iodobenzene (PIFA) in the reaction (Entry 1 and 6).

Compared to previous work on the synthesis of triazole-spirodiones [8], our current approach shortens the synthesis steps and broadens the range of substituents on nitrogen. For example, in the reaction step converting triazoles to alkanes, a variety of heterocyclic or aromatic rings derivatives can be obtained in fewer synthetic steps. At the same time, R_1_ can be varied to increase the application range of the substrate.

Next, the effect of different bases was investigated, including potassium carbonate (K_2_CO_3_), sodium hydride (NaH), 1,8-diazabicyclo[5.4.0]undec-7-ene (DBU), 1,4-diazabicyclo[2.2.2]octane (DABCO), and trimethylamine (Et_3_N) on the alkylation of 1-oxa-4-azaspiro[4.5] deca-6,9-diene-3,8-dione (**10a**) with propargyl bromide. DBU was the best available base for the reaction based on the results in Table 2.

**Scheme 2:**
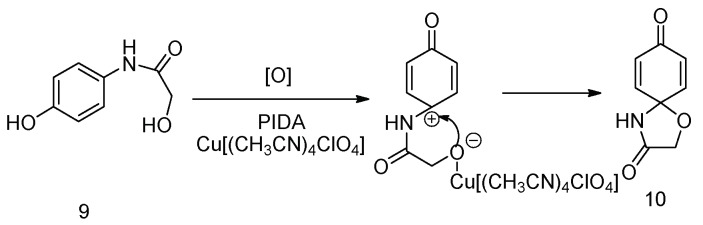
Proposed copper-catalyzed oxidation mechanism.

Based on literature reports [19,20,21,22,23,24], the PIDA oxidation step may have proceeded by a mechanism where a copper-conjugated intermediate is formed as illustrated in Scheme 2. In the presence of PhI(OAc)2, 2-hydroxyn-(4-hydroxyphenyl) acetamide is oxidized to quinone and then converted to a carbon cation. At the same time, the hydroxyl group forms a complex with copper, so that the oxygen of hydroxyl can facilitate the attack to the carbon cation, thereby closing the ring to form a spiral ring derivative.

### 2.3. Antitumor Activity of 1-Oxa-4-azaspiro[4.5]deca-6,9-diene-3,8-diones

Next, we investigated in vitro antitumor activities of the synthesized 1-oxa-4-azaspiro[4.5] deca-6,9-diene-3,8-dione derivatives toward lung cancer cell line A549, breast cancer cell line MDA-MB-231, and cervical cancer cell line HeLa with bendamustine (a bifunctional alkylating agent) and vorinostat (an HDAC inhibitor) as positive drug controls (Table 3). All the compounds except **12a** showed more potent antitumor activity against MDA-BM-231 and HeLa cell lines than bendamustine and vorinostat. Among them, compounds **11b** and **11h** had IC_50_ values of 0.18 and 0.19 μM against A549, respectively; the IC_50_ values of **11d**, **11h**, and **11k** were 0.09, 0.08, and 0.08 μM toward MDA-BM-231, respectively; and the compounds with IC_50_ value below 0.20 μM against HeLa cells were **11f**, **11h**, **11k**, and **12c**. Compound **11h** emerged as a promising candidate for further research, showing most antiproliferative efficacy overall against all three cancer cell lines. From the activity test of this series of derivatives, it can be seen that 1-oxa-4-azaspiro[4.5]indole-6,9-diene-3,8-dione is an active essential group. Secondly, from the antitumor data of **10a** ≤ **10b**, **11c** ≤ **11j**, **11h** ≤ **11k**, it can be seen that the activity of 2-CH_3_ decreased slightly. When the 4-position substituent is *p*-bromobenzyl, the activity appears to be optimal.

## 3. Materials and Methods

### 3.1. Instruments and Reagents

NMR spectra were recorded on a Mercury 400 MHz NMR, Varian, Palo Alto, CA, USA and an 600 MHz NMR, Agilent Technologies Inc. Palo Alto, California, USA(CDCl3 was the solvent and TMS was the internal standard). MS spectra were measured on Bruker Daltonics Data Analysis 3.4 Mass Spectrometer, Bruker, Karlsruhe, Germany and Thermo LTQ Orbitrap-XL Mass Spectrometer (Thermo Scientific, Waltham, MA, USA). A YRT-3 melting point instrument (Tianda Tianfa Company, Tianjin, China) was used for measuring melting points, where the measured temperature was uncorrected. HSGF 254 high-efficiency thin-layer chromatography silica gel plates were purchased from Huiyou Development Co., Yantai, Shangdong, China. Ltd. HSGF 254 thin-layer silica gel (300 mesh ~ 400 mesh) was purchased from Ocean Chemical Plant (Qingdao, Shangdong, China). The reagents used were analytically pure unless otherwise specified, and the solvents used were dried by conventional methods. (NMR spectra (1H and 13C) of all 17 1-oxa-4-azaspiro[4.5] deca-6,9-diene-3,8-dione derivatives are provided in Supplementary Materials).

### 3.2. Synthesis of 1-Oxa-4-azaspiro[4.5]deca-6,9-diene-3,8-diones

#### 3.2.1. Synthesis of Compounds **10a**–**10b**

In a 25 mL single-neck round bottom flask and under a nitrogen atmosphere, compounds **9a** or **9b** (1 mmol), PhI(OAc)_2_ (2 mmol), and Cu[(CH_3_CN)_4_ClO_4_] (0.05 mmol) were added to dried DCM (10 mL), and the reaction mixture was stirred at room temperature. The reaction was monitored by thin layer chromatography (TLC) (petroleum ether/ethyl acetate = 1:1). After completion of the reaction, the mixture was concentrated under reduced pressure and purified by column chromatography.

*1-Oxa-4-azaspiro[4.5]deca-6,9-diene-3,8-dione* (**10a**): 119 mg viscous off-white solid, yield 72%; ^1^H NMR (600 MHz, CDCl_3_) *δ* 7.77 (s, 1H), 6.72 (d, *J* = 8.8 Hz, 2H), 6.22 (d, *J* = 8.8 Hz, 2H), 4.36 (s, 2H). ^13^C NMR (150 MHz, CDCl_3_) *δ* 183.9, 172.8, 143.9, 129.6, 83.8, 66.2. HR-MS (ESI) *m/z*: calcd C_8_H_7_NO_3_ [M + H]^+^ 166.0504, found 166.0502.

*2-Methyl-1-oxa-4-azaspiro[4.5]deca-6,9-diene-3,8-dione* (**10b**): 130 mg viscous off-white solid, yield 73%; ^1^H NMR (400 MHz, CDCl_3_) *δ* 7.80 (s, 1H), 6.70 (ddd, *J* = 43.1, 10.0, 3.1 Hz, 2H), 6.23 (ddd, *J* = 14.6, 10.2, 1.9 Hz, 2H), 4.53 (q, *J* = 6.7 Hz, 1H), 1.49 (d, *J* = 6.7 Hz, 3H). ^13^C NMR (100 MHz, CDCl_3_) *δ* 184.2, 175.2, 145.5, 144.4, 130.2, 129.0, 82.2, 73.3, 18.4. HRMS (ESI) calcd C_9_H_9_NO_3_ [M + H]^+^ 180.0661, found 180.0658.

#### 3.2.2. Synthesis of Compounds **11a**–**11k**

In a 10 mL one-necked round bottom flask, compound **3** (0.1 mmol), dry THF (2 mL), and DBU (0.15 mmol) were added, and the mixture was stirred for 10 min in an ice water bath, then the halogenated hydrocarbon (0.12 mmol) was slowly added. The reaction was monitored by chromatography (TLC) (petroleum ether/ethyl acetate = 2:1). After completion of the reaction, a saturated NH_4_Cl solution was added and extracted with ethyl acetate. The organic layer was separated, dried with MgSO4, and evaporated to obtain crude products which was purified by flash chromatography.

*4-Methyl 1-oxa-4-azaspiro[4.5]deca-6,9-diene-3,8-dione* (**11a**): 13 mg white solid, yield 74%, mp 112–114 °C; ^1^H NMR (400 MHz, CDCl_3_) *δ 6*.60 (d, *J* = 10.1 Hz, 2H), 6.36 (d, *J* = 10.0 Hz, 2H), 4.43 (s, 2H), 2.74 (s, 3H). ^13^C NMR (100 MHz, CDCl_3_) *δ* 183.7, 169.4, 143.4, 131.5, 87.5, 66.6, 25.1. HRMS (ESI) calcd C_9_H_9_NO_3_ [M + H]^+^ 180.0661, found 180.0658.

*4-Ethyl-1-oxa-4-azaspiro[4.5]deca-6,9-diene-3,8-dione* (**11b**): 17 mg of white solid, yield 86%, mp 93–96 °C; ^1^H NMR (400 MHz, CDCl_3_) *δ* 6.66–6.60 (m, 2H), 6.37–6.28 (m, 2H), 4.39 (s, 2H), 3.21 (q, *J* = 7.2 Hz, 2H), 1.16 (t, *J* = 7.2 Hz, 3H). ^13^C NMR (100 MHz, CDCl_3_) *δ* 183.9, 169.5, 144.1, 130.9, 87.5, 66.5, 35.1, 14.6. HRMS (ESI) calcd C_10_H_11_NO_3_ [M + H]^+^ 194.0817, found 194.0815.

*4-(Prop-2-yn-1-yl)-1-oxa-4-azaspiro[4.5]deca-6,9-diene-3,8-dione* (**11c**): 14 mg of white solid. Yield 67%, mp 155–157 °C; ^1^H NMR (400 MHz, CDCl_3_) *δ* 6.68 (d, *J* = 10.1 Hz, 2H), 6.35 (d, *J* = 10.1 Hz, 2H), 4.43 (s, 2H), 3.99 (d, *J* = 2.5 Hz, 2H), 2.22 (t, *J* = 2.5 Hz, 1H). ^13^C NMR (150 MHz, CDCl_3_) *δ* 183.7, 168.7, 143.1, 131.5, 87.1, 77.3, 73.1, 66.2, 28.8. HRMS (ESI) calcd C_11_H_9_NO_3_ [M + H]^+^ 203.0582, found 203.0580.

*4-(3-Methylbut-2-en-1-yl)-1-oxa-4-azaspiro[4.5]deca-6,9-diene-3,8-dione* (**11d**): 17mg brown liquid, yield 74%: ^1^H NMR (400 MHz, CDCl_3_) *δ* 6.62 (d, *J* = 10.1 Hz, 2H), 6.29 (d, *J* = 10.1 Hz, 2H), 4.41 (s, 2H), 3.81 (d, *J* = 7.2 Hz, 2H), 1.65 (s, 3H), 1.53 (s, 3H). ^13^C NMR (150 MHz, CDCl_3_) *δ* 184.1, 169.3, 144.2, 137.4, 130.4, 118.8, 87.2, 66.5, 37.8, 25.5, 18.0. HRMS (ESI) calcd C_13_H_15_NO_3_ [M + H]^+^ 234.1130, found 234.1128.

*4-(But-2-yn-1-yl)-1-oxa-4-azaspiro[4.5]deca-6,9-diene-3,8-dione* (**11e**): 17 mg yellow-white solid, yield 76%, mp 77–79 °C; ^1^H NMR (400 MHz, CDCl_3_) *δ* 6.67 (d, *J* = 10.1 Hz, 2H), 6.34 (d, *J* = 10.1 Hz, 2H), 4.41 (s, 2H), 3.94 (d, *J* = 2.3 Hz, 2H), 1.72 (t, *J* = 2.3 Hz, 3H). ^13^C NMR (150 MHz, CDCl_3_) *δ* 184.0, 169.0, 143.5, 131.1, 87.1, 81.2, 72.8, 66.3, 29.3, 3.4. HRMS (ESI) calcd C_12_H_11_NO_3_ [M + H]^+^ 218.01717, found 218.0815.

*(E)-4-(3,7-Dimethyloctyl-2,6-dien-1-yl)-1-oxa-4-azaspiro[4.5]deca-6,9-diene-3,8-dione* (**11f**): 21 mg of a brown liquid, yield 70%; ^1^H NMR (400 MHz, CDCl_3_) *δ* 6.60 (d, *J* = 10.0 Hz, 2H), 6.26 (d, *J* = 10.0 Hz, 2H), 5.06 (s, 1H), 5.01 (t, *J* = 6.6 Hz, 1H), 4.40 (s, 2H), 3.82 (d, *J* = 7.1 Hz, 2H), 2.04–1.99 (m, 2H)), 1.95 (d, *J* = 7.4 Hz, 2H), 1.67 (s, 3H), 1.58 (s, 3H), 1.51 (s, 3H). ^13^C NMR (100 MHz, CDCl_3_) *δ* 184.1, 169.3, 144.2, 140.8, 131.9, 130.5, 123.6, 118.7, 87.2, 66.6, 39.3, 37.7, 26.0, 25.7, 17.7, 16.4. HRMS (ESI) calcd C_18_H_23_NO_3_ [M + H]^+^ 302.1756, found 302.1755.

*4-(4-Methylbenzyl)-1-oxa-4-azaspiro[4.5]deca-6,9-diene-3,8-dione* (**11g**): 22 mg viscous white solid, yield 81%, mp 76–78 °C; ^1^H NMR (400 MHz, CDCl_3_) *δ* 7.06 (d, *J* = 1.8 Hz, 4H), 6.48–6.39 (m, 2H), 6.17–6.11 (m, 2H), 4.46 (s, 2H), 4.35 (s, 2H), 2.31 (s, 3H). ^13^C NMR (100 MHz, CDCl_3_) *δ* 184.0, 169.7, 143.8, 138.0, 133.1, 130.6, 129.3, 128.6, 87.4, 66.4, 43.5, 21.1. HRMS (ESI) calcd C_16_H_15_NO_3_ [M + Na]^+^ 292.0950, found 292.0944.

*4-(4-Bromobenzyl)-1-oxa-4-azaspiro[4.5]deca-6,9-diene-3,8-dione* (**11h**): 25 mg of light yellow liquid, yield 75%, mp 90–93 °C; ^1^H NMR (400 MHz, CDCl_3_) *δ* 7.41 (d, *J* = 8.1 Hz, 2H), 7.06 (d, *J* = 8.1 Hz, 2H), 6.43 (d, *J* = 9.9 Hz, 2H), 6.18 (d, *J* = 9.9 Hz, 2H), 4.47 (s, 2H), 4.33 (s, 2H). ^13^C NMR (100 MHz, CDCl_3_) *δ* 183.8, 169.9, 143.5, 135.2, 131.9, 130.9, 130.3, 122.3, 87.3, 66.3, 43.1. HRMS (ESI) calcd C_15_H_12_BrNO_3_ [M + Na]^+^ 355.9898, found 355.9892.

*4-(4-Nitrobenzyl)-1-oxa-4-azaspiro[4.5]deca-6,9-diene-3,8-dione* (**11i**): 11 mg viscous off-white solid, yield 36%; ^1^H NMR (400 MHz, CDCl_3_) *δ* 8.26 (d, *J* = 8.4 Hz, 2H), 7.57 (d*, J* = 8.3 Hz, 2H), 6.59 (d, *J* = 9.8 Hz, 2H), 6.20 (d, *J* = 9.8 Hz, 2H), 5.42 (s, 2H), 4.67 (s, 2H). ^13^C NMR (150 MHz, CDCl_3_) *δ* 185.1, 170.7, 145.1, 142.0, 128.6, 128.4, 124.0, 119.2, 97.8, 68.7, 31.6. HRMS (ESI) calcd C_15_H_12_N_2_O_5_ [M + H]^+^ 301.0824, found 301.0822.

*2-Methyl-4-(prop-2-yn-1-yl)-1-oxa-4-azaspiro[4.5]deca-6,9-diene-3,8-dione* (**11j**): 13 mg of off-white solid, yield 60%, mp 153–155 °C; ^1^H NMR (400 MHz, CDCl_3_) *δ* 6.68 (dd, *J* = 10.0, 2.8 Hz, 1H), 6.61 (dd, *J* = 10.0, 2.8 Hz, 1H), 6.34 (dd, *J* = 14.8, 10.1 Hz, 2H), 4.56 (q, *J* = 6.7 Hz, 1H), 4.05–3.91 (m, 3H), 1.51 (d, *J* = 6.7 Hz, 3H). ^13^C NMR (150 MHz, CDCl_3_) *δ* 183.9, 171.3, 144.6, 143.3, 131.9, 130.8, 85.5, 77.4, 73.1, 73.1, 28.9, 18.5. HRMS (ESI) calcd C_12_H_11_NO_3_ [M + H]^+^ 218.0817, found 218.0817.

*4-(4-Bromobenzyl)-2-methyl-1-oxa-4-azaspiro[4.5]deca-6,9-diene-3,8-dione* (**11k**): 23 mg viscous yellow-white solid, yield 67%; ^1^H NMR (400 MHz, CDCl_3_) *δ* 7.40 (d, *J* = 8.3 Hz, 2H), 7.05 (d, *J* = 8.2 Hz, 2H), 6.44 (dd, *J* = 10.0, 3.0 Hz, 1H), 6.33 (dd, *J* = 10.0, 3.0 Hz, 1H), 6.26–6.05 (m, 2H), 4.60 (q, *J* = 6.7 Hz, 1H), 4.41–4.23 (m, 2H), 1.53 (d, *J* = 6.7 Hz, 3H). ^13^C NMR (100 MHz, CDCl_3_) *δ* 183.9, 172.2, 145.1, 143.7, 135.4, 131.9, 131.4, 130.3, 130.2, 122.2, 85.7, 73.2, 43.3, 18.7. HRMS (ESI) calcd C_16_H_15_BrNO_3_ [M + H]^+^ 301.0824, found 301.0822.

#### 3.2.3. Synthesis of Compounds **12a**–**12d**

To an anhydrous DMSO (1 mL) solution of NaN_3_ (0.5 mmol) was added organic halide (0.5 mmol) and the mixture was stirred overnight. Water (2–3 mL) was added, followed by solid sodium ascorbate (0.05 mol), the alkyne **11c** or **11j** (0.5 mmol), and aqueous CuSO_4_ solution (1mL, 1 M). The mixture was stirred for 3–12 h until the starting material disappeared, then more water was added slowly until the product precipitated completely from the solution. The product was collected by filtration, washed with water, and dried in air.

(2*R*,3*R*,4*S*,5*R*,6*S*)-2-(Acetoxymethyl)-6-(4-((3,8-dioxo-1-oxa-4-azaspiro[4.5]deca-6,9-dien-4yl)methyl)-1H-1,2,3-triazol-1-yl)tetrahydro-2H-pyran-3,4,5-triyl triacetate (**12a**): 31 mg of white solid, yield 54%, mp 183–185 °C; ^1^H NMR (400 MHz, CDCl_3_) *δ* 7.81 (s, 1H), 6.53 (ddd, *J* = 34.7, 10.1, 3.2 Hz, 2H), 6.26 (ddd, *J* = 62.1, 10.1, 2.2 Hz, 2H), 5.81 (d*, J* = 9.2 Hz, 1H), 5.42 (t, *J* = 9.5 Hz, 1H), 5.26 (dt, *J* = 29.9, 9.6 Hz, 2H), 4.57 (d, *J* = 15.7 Hz, 1H), 4.45 (s, 2H), 4.40–4.29 (m, 2H), 4.19–4.12 (m, 1H), 3.99 (ddd, *J* = 10.2, 5.0, 2.2 Hz, 1H), 2.10 (s, 3H)), 2.06 (s, 3H), 2.03 (s, 3H), 1.89 (s, 3H). ^13^C NMR (150 MHz, CDCl_3_) *δ* 183.7, 170.5, 169.8, 169.3, 168.9, 143.5, 143.4, 143.1, 131.6, 130.9, 121.6, 87.3, 85.9, 75.2, 72.3, 70.6, 67.6, 66.2, 61.4, 35.0, 20.7, 20.5, 20.5, 20.1. HRMS (ESI) calcd C_25_H_28_N_4_O_12_ [M + H]^+^ 577.1782, found 577.1776.

*4-((1-Ethyl-1H-1,2,3-triazol-4-yl)methyl)-1-oxa-4-azaspiro[4.5]deca-6,9-diene-3,8-dione* (**12b**): 16 mg, yellow-white viscous liquid, yield 58%; ^1^H NMR (400 MHz, CDCl_3_) *δ* 7.51 (d*, J* = 8.4 Hz, 2H), 7.46 (s, 1H), 7.12 (d, *J* = 8.4 Hz, 2H), 6.50 (ddd, *J* = 17.9, 10.0, 3.1 Hz, 2H), 6.28 (dd, *J* = 10.0, 2.1 Hz, 1H), 6.19 (dd, *J* = 10.0, 2.1 Hz, 1H), 4.54 (q*, J* = 6.7 Hz, 1H), 4.41 (s, 2H), 1.48 (d, *J* = 6.7 Hz, 3H). ^13^C NMR (100 MHz, CDCl_3_) *δ* 183.8, 169.8, 143.4, 142.3, 131.2, 122.5, 87.5, 66.3, 45.4, 35.2, 15.4. HRMS (ESI) calcd C_13_H_14_N_4_O_3_ [M + H]^+^ 275.1144, found 275.1140.

*4-((1-(4-Bromobenzyl)-1H-1,2,3-triazol-4-yl)methyl)-2-methyl-1-oxa-4-azaspiro[4.5]deca-6,9-diene-3,8-dione* (**12c**): 21 mg of yellow-white viscous liquid, yield 49%; ^1^H NMR (400 MHz, CDCl_3_) *δ* 7.53–7.49 (m, 2H), 7.46 (s, 1H), 7.12 (d, *J* = 8.4 Hz, 2H), 6.55–6.45 (m, 2H), 6.28 (dd, *J* = 10.0, 2.1 Hz, 1H), 6.19 (dd, *J* = 10.0, 2.1 Hz, 1H), 4.54 (q*, J* = 6.7 Hz, 1H), 4.41 (s, 2H), 1.48 (d, *J* = 6.7 Hz, 3H). ^13^C NMR (100 MHz, CDCl_3_) *δ* 183.9, 172.1, 144. 8,143.5, 142.9, 133.3, 132.4, 131.7, 130.5, 129.7, 123.1, 123.1, 85.8, 73.2, 35.3, 18.6. HRMS (ESI) calcd C_19_H_17_BrN_4_O_3_ [M + Na]^+^ 451.0382, found 451.0378.

(2*R*,3*R*,4*S*,5*R*,6*S*)-2-(Acetoxymethyl)-6-(4-((2-methyl-3,8-dioxo-1-oxa-4-azaspiro[4.5]deca-6,9-dien-4-yl) methyl)-1H-1,2,3-triazol-1-yl)tetrahydro-2H-pyran-3,4,5-triyl triacetate (**12d**): 27 mg of white solid, yield 47%, mp 86–90 °C; ^1^H NMR (400 MHz, CDCl_3_) *δ* 7.81 (s, 1H), 6.62–6.05 (m, 4H), 5.81 (d, *J* = 9.2 Hz, 1H), 5.41 (td, *J* = 9.5, 1.9 Hz, 1H), 5.31–5.19 (m, 2H), 4.58 (t, *J* = 15.7 Hz, 2H), 4.35–4.25 (m, 2H), 4.14 (d*, J* = 12.4 Hz, 1H), 4.03–3.95 (m, 1H), 2.09 (s, 3H), 2.05 (s, 3H), 2.01 (s, 3H), 1.87 (s, 3H), 1.51 (t*, J* = 6.8 Hz, 3H). ^13^C NMR (150 MHz, CDCl_3_) *δ* 183.8 (2C), 172.09, 172.1, 170.5, 169.8 (2C), 169.3 (169.32), 169.3 (169.29), 168.9, 168.8, 144.9, 144.6, 143.7, 143.6, 143.5, 143.3, 132.1, 131.3, 131.0, 130.2, 121.65(2C), 85.9(85.88), 85.9(85.85), 85.8, 85.7, 75.2, 75.0, 73.2, 73.1, 72.2 (2C), 70.7, 70.6, 69.2, 67.6 (2C), 61.4, 59.1, 35.2 (2C), 20.7 (2C), 20.5 (20.49), 20.5 (20.46), 20.1 (20.13), 20.1 (20.11), 18.6, 18.4, 16.5 (2C). HRMS (ESI) calcd for C_26_H_30_N_4_O_12_ [M + H]^+^ 591.1938, found 591.1933.

### 3.3. Cell Culture and Antiproliferative Assays

Human non-small cell lung carcinoma (A549), human breast adenocarcinoma (MDA-MB-231), and human cervix carcinoma (HeLa) cell lines (ATCC, Manassas, VA, USA) were grown in DMEM supplemented with 115 units/mL of penicillin G, 115 μg/mL of streptomycin, and 10% fetal bovine serum (all from Life Technologies, Grand Island, NY, USA). Cells were seeded in 96-well plates (5 × 10^3^ cells/well) containing 50 μL growth medium for 24 h. After medium removal, 100 μL fresh medium containing individual compounds and both bendamustine and vorinostat controls at different concentrations was added to each well and incubated at 37 °C for 72 h. After 24 h of culture, the cells were supplemented with 50 μL of compounds, bendamustine, or vorinostat dissolved in DMSO (less than 0.25% in each preparation). After 72 h of incubation, 20 μL of resazurin was added for 2 h before recording fluorescence at 560 nm (excitation) and 590 nm (emission) using a VICTOR microtiter plate fluorometer (Perkin-Elmer, Waltham, MA, USA). The IC_50_ was defined as the compound concentration required to inhibit cell proliferation by 50% in comparison with cells treated with the maximum amount of DMSO (0.25%) which was considered as 100% viability.

## 4. Conclusions

From 4-aminophenol and α-glycolic acid or lactic acid, a series of novel 1-oxa-4- azaspiro[4.5]deca-6,9-diene-3,8-dione derivatives were synthesized through a newly developed approach including a key intramolecular metal-catalyzed oxidative cyclization. The preliminary antitumor activity of 17 1-oxa-4-azaspiro[4.5]deca-6,9-diene-3,8-dione derivatives was investigated, and the results indicate that all the compounds showed moderate to potent antitumor activity against A549, MDA-BM-231, and HeLa cell lines. The most potent compound, **11h**, has IC_50_ values of 0.19, 0.08, and 0.15 µM against A549, MDA-BM-231, and HeLa cell lines, respectively, which is a promising candidate for further research.

## Figures and Tables

**Table 1:** Optimization of the reaction conditions for the formation of compound **10a**.

Entry	Oxidizing Agent (2 eq)	Metal Catalyst (0.05 eq)	Yield (%)
1	PhI(OAc)_2_	Cu[(CH_3_CN)_4_ClO_4_]	72
2	PhI(OAc)_2_	Rh_2_(OAc)_4_	75
3	PhI(OAc)_2_	Mn(OAc)_2_	trace
4	PhI(OAc)_2_	FeCl_2_	trace
5	PhI(OAc)_2_	ZnCl_2_	trace
6	PIFA	Cu[(CH_3_CN)_4_ClO_4_]	68
7	PhI(OAc)_2_	No	trace

**Table 2:** Optimization of the reaction conditions for compound **11c**.

Entry	Base (1.5 eq)	Temperature (°C)	Yield (%)
1	K_2_CO_3_	25	trace
2	NaH	0	50
3	DBU	0-25	67
4	DABCO	80	trace
5	Et_3_N	25	trace
6	No	25	no reaction

**Table 3:** In vitro antitumor activities of 1-oxa-4-azaspiro[4.5]deca-6,9-diene-3,8-diones against cancer cell lines.

Compound	IC_50_ (μM)		
	A549	MDA-BM-231	HeLa
**10a**	0.23 ± 0.15	0.28 ± 0.07	0.26 ± 0.12
**10b**	0.52 ± 0.27	0.18 ± 0.08	1.34 ± 0.28
**11a**	0.34 ± 0.09	0.23 ± 0.06	0.47 ± 0.13
**11b**	0.18 ± 0.01	0.12 ± 0.06	0.27 ± 0.15
**11c**	0.37 ± 0.11	0.13 ± 0.07	0.22 ± 0.05
**11d**	0.72 ± 0.09	0.09 ± 0.02	0.20 ± 0.05
**11e**	0.29 ± 0.10	0.15 ± 0.04	0.31 ± 0.16
**11f**	0.24 ± 0.08	0.11 ± 0.05	0.19 ± 0.12
**11g**	0.26 ± 0.14	0.10 ± 0.03	0.20 ± 0.07
**11h**	0.19 ± 0.03	0.08 ± 0.02	0.15 ± 0.02
**11i**	0.81 ± 0.13	0.21 ± 0.11	0.27 ± 0.14
**11j**	0.39 ± 0.14	0.17 ± 0.06	0.60 ± 0.12
**11k**	0.26 ± 0.08	0.08 ± 0.004	0.14 ± 0.07
**12a**	>10	4.75 ± 0.90	>10
**12b**	0.76 ± 0.10	0.27 ± 0.04	0.41 ± 0.04
**12c**	0.90 ± 0.03	0.31 ± 0.01	0.14 ± 0.1
**12d**	5.31 ± 1.01	0.31 ± 0.08	1.65 ± 0.46
bendamustine	-	13.28 ± 0.53	>20
vorinostat	-	3.62 ± 0.18	4.52 ± 0.27

± standard deviation (SD) of triplicate experiments.

## Supplementary Materials

The NMR spectra (^1^H and ^13^C) of all 17 1-oxa-4-azaspiro[4.5] deca-6,9-diene-3,8-dione derivatives **10a**–**10b**, **11a**–**11k**, and **12a**–**12d** are available online.
