# Supplementary material for: Synthesis and Antitumor Activity of a Series of Novel 1-Oxa-4-azaspiro[4,5]deca-6,9-diene-3,8-dione Derivatives
Source: Molecules. 2019 Mar 7;24(5):936. doi: 10.3390/molecules24050936 (PMC6429447; doi:10.3390/molecules24050936)
Supplement: Supplementary file 1 [file molecules-24-00936-s001.pdf]

Supplementary Materials

Synthesis and Antitumor Activity of a Series of Novel 1-Oxa-4-azaspiro[4,5]deca-6,9-diene-3,8-dione Derivatives

Ze Yang [1,2], Qiu Zhong [3], Shilong Zheng [3], Guangdi Wang [3,*] and Ling He [1,*]

1 Key Laboratory of Drug–Targeting and Drug Delivery System of the Education Ministry, Department of Medicinal Chemistry, West China School of Pharmacy, Sichuan University, Chengdu, Sichuan 610041, China; 2016224055105@stu.scu.edu.cn

2 Sichuan Engineering Laboratory for Plant–Sourced Drug and Sichuan Research Center for Drug Precision Industrial Technology, Department of Medicinal Chemistry, West China School of Pharmacy, Sichuan University, Chengdu, Sichuan 610041, China

3 Department of Chemistry, RCMI Cancer Research Center, Xavier University of Louisiana, New Orleans, LA 70125, USA; qzhong@xula.edu (Q.Z.); szheng@xula.edu (S.Z.)

* Correspondence: gwang@xula.edu (G.W.); heling2012@scu.edu.cn. (L.H.)

(Supporting Information)

^{1}H and ^{13}C NMR spectra of compounds **10a–10b**---S2–S3

^{1}H and ^{13}C NMR spectra of compounds **11a–11k**---S4–S14

^{1}H and ^{13}C NMR spectra of compounds **12a–12d**---S15–S18

Compound **10a**

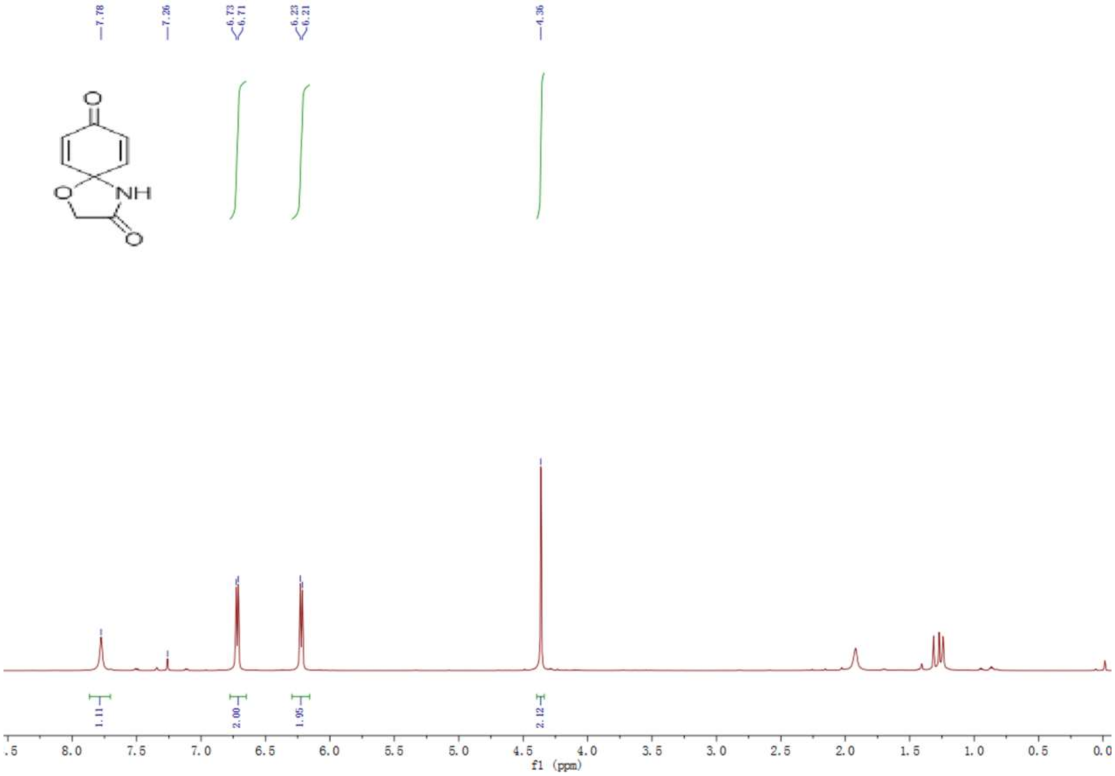

^{1}H NMR spectra of compound **10a**

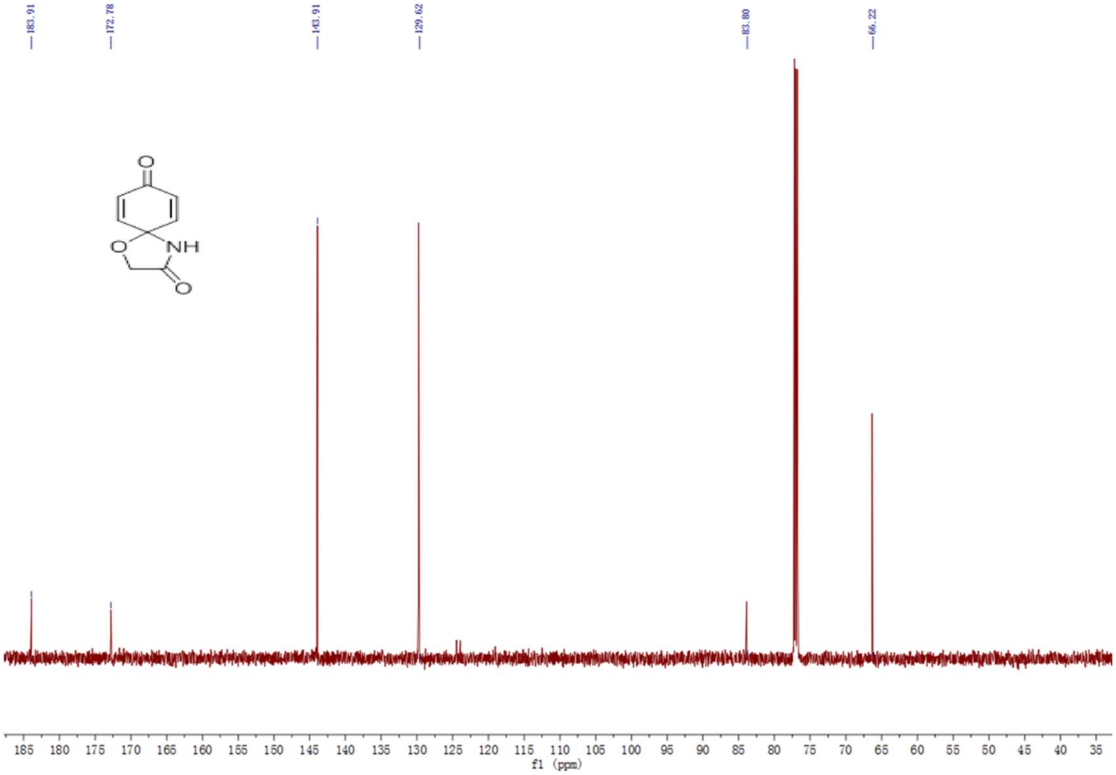

^{13}C NMR spectra of compound **10a**

Compound **10b**

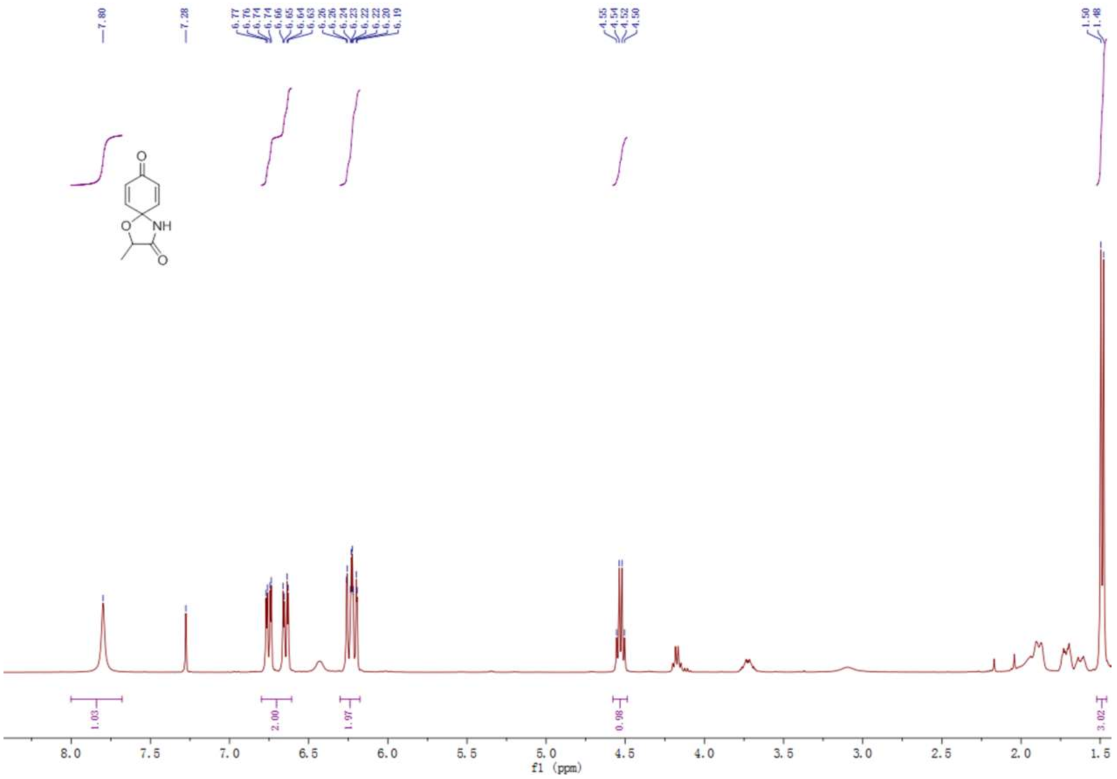

^{1}H NMR spectra of compound **10b**

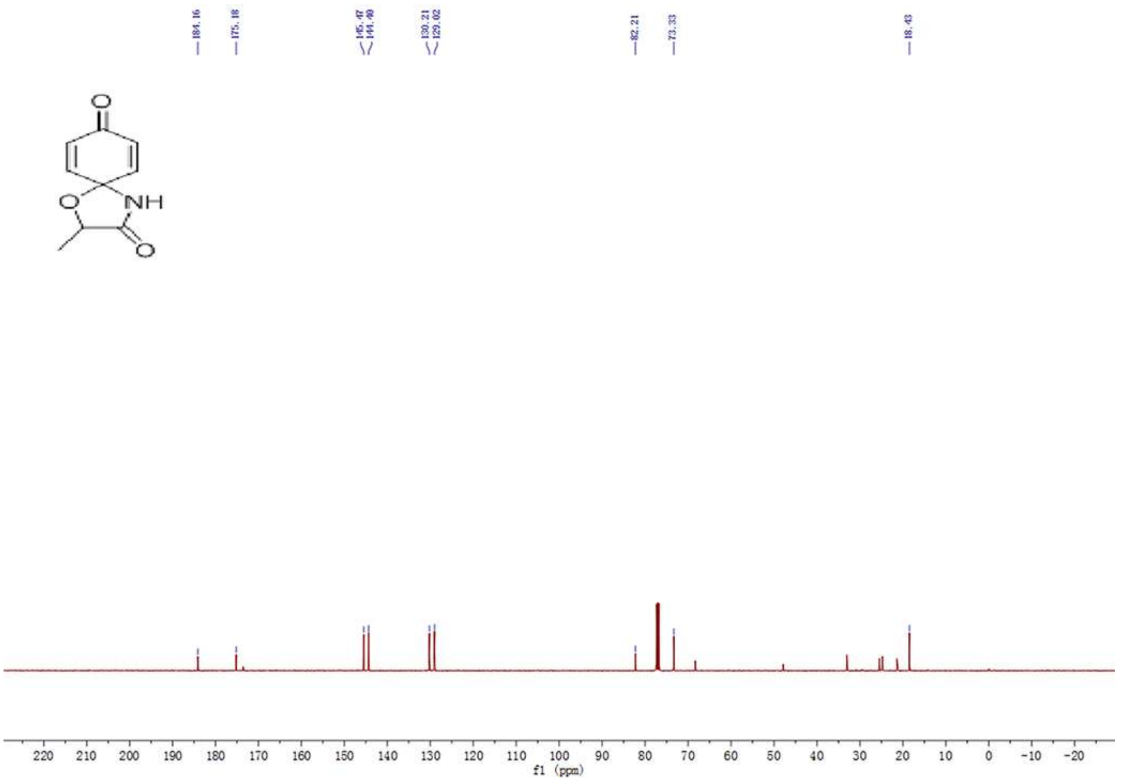

^{13}C NMR spectra of compounds **10b**

Compound **11a**

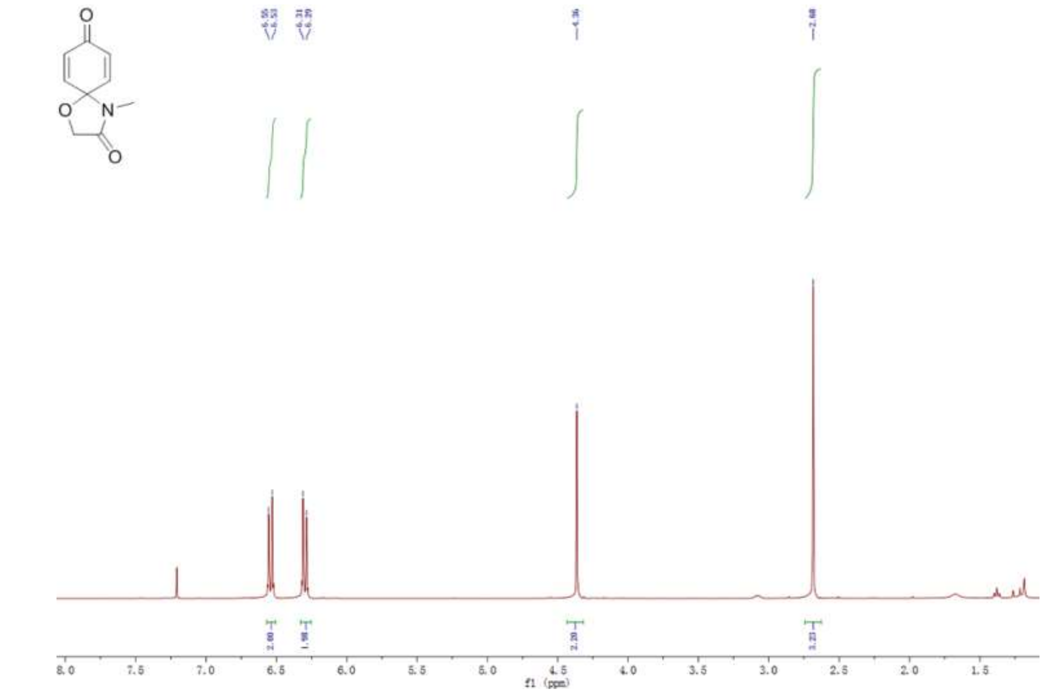

^{1}H NMR spectra of compound **11a**

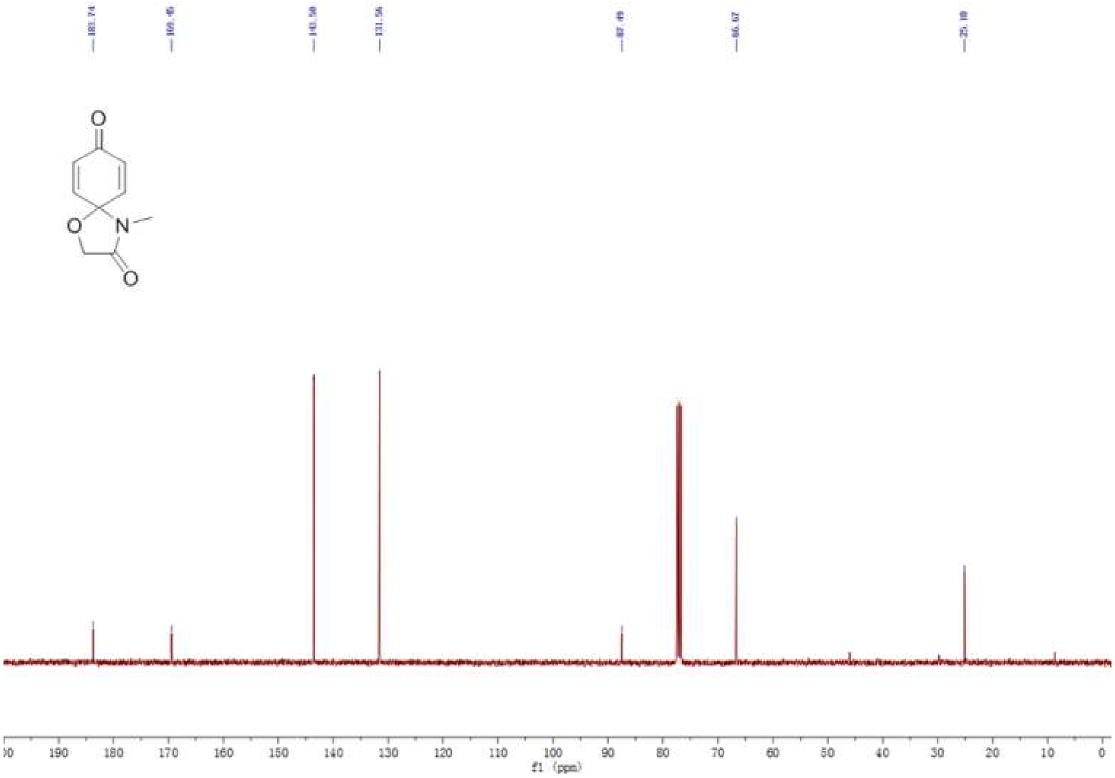

^{13}C NMR spectra of compounds **11a**

Compound **11b**

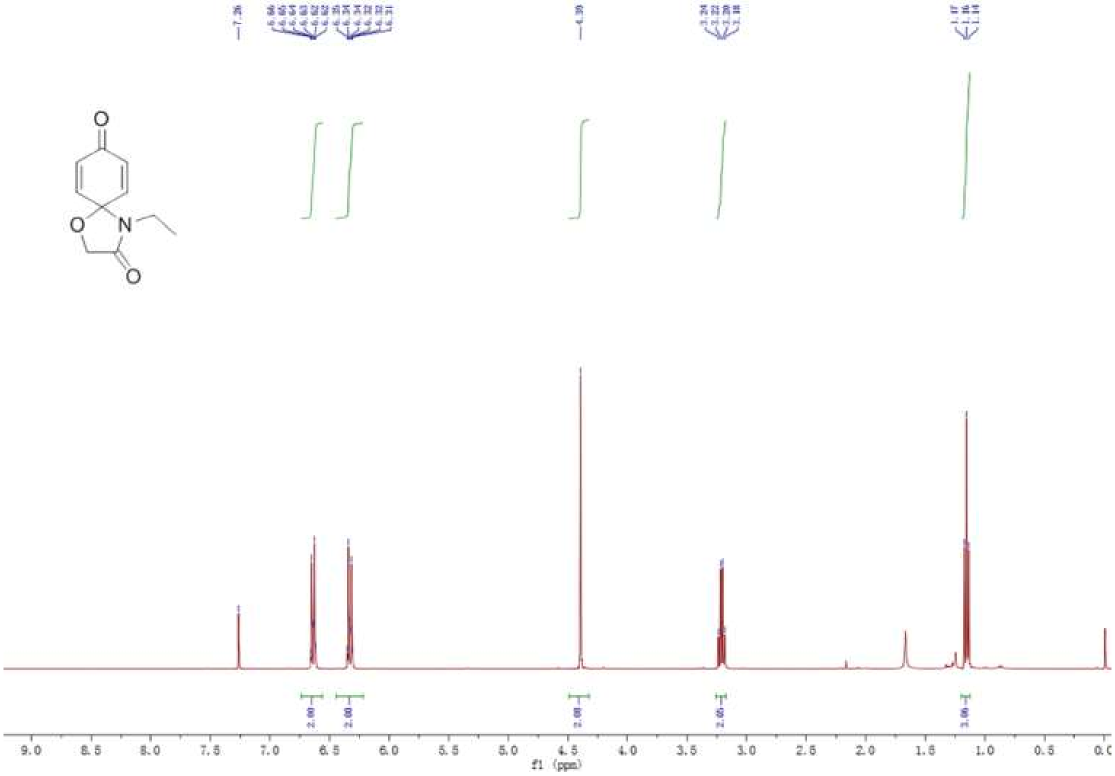

^{1}H NMR spectra of compound **11b**

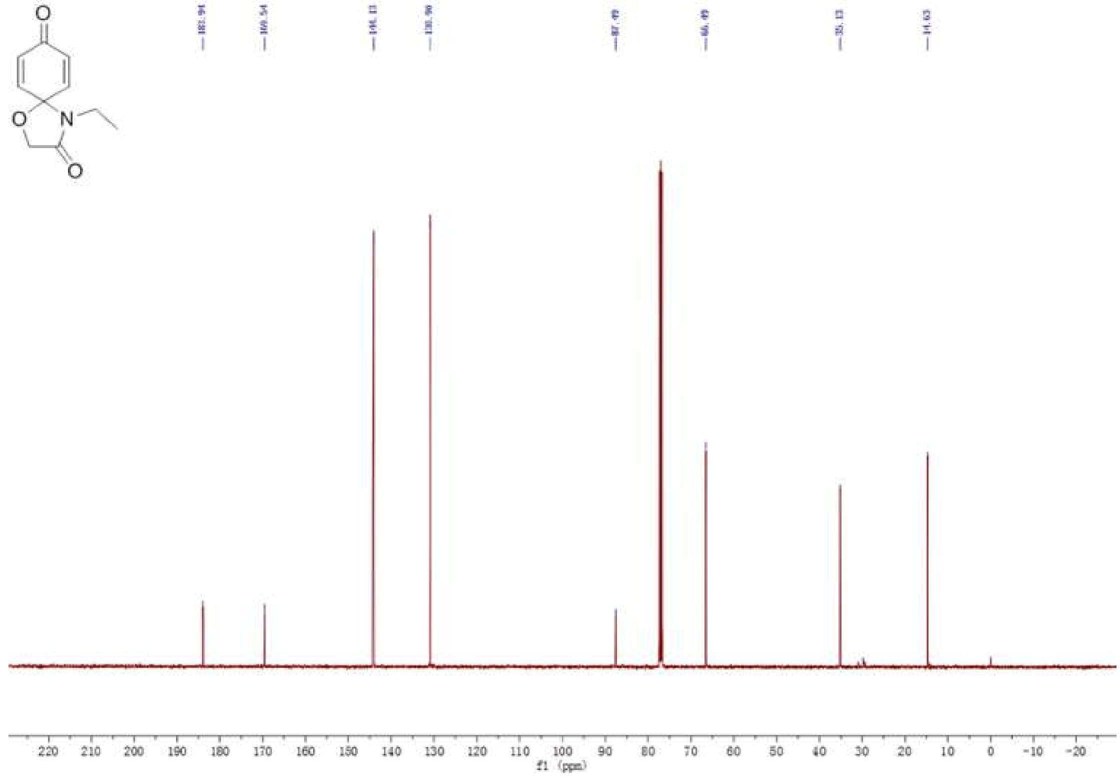

^{13}C NMR spectra of compounds **11b**

Compound **11c**

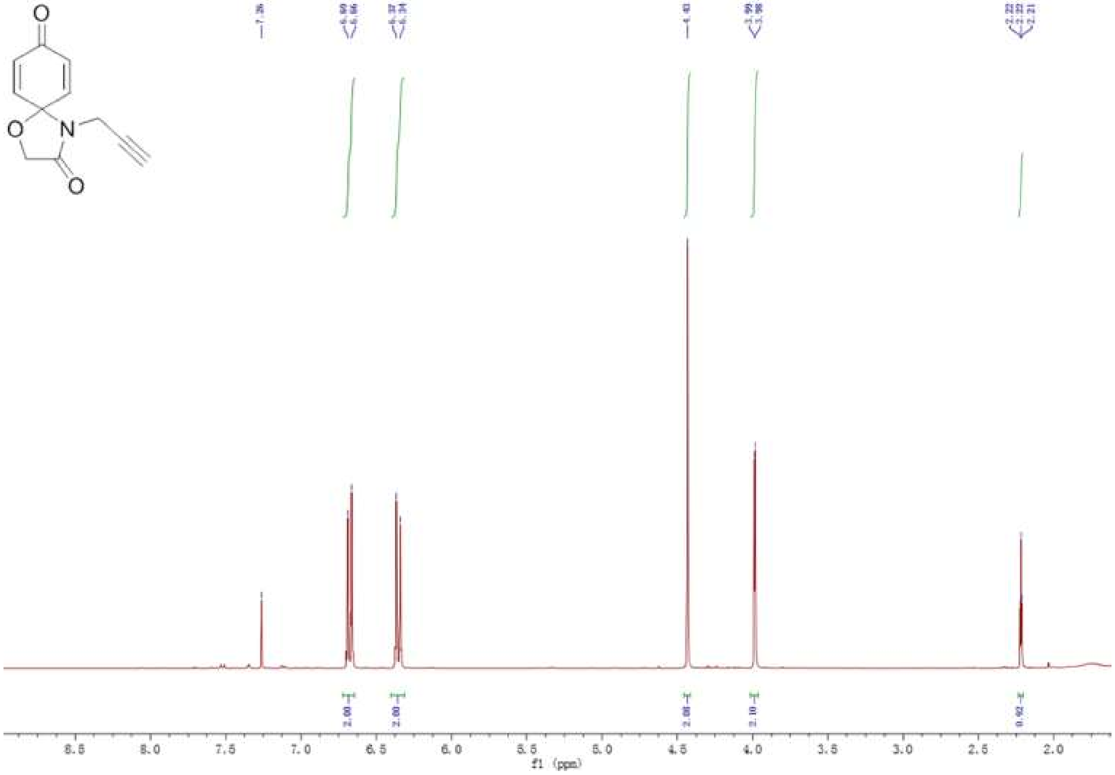

^{1}H NMR spectra of compound **11c**

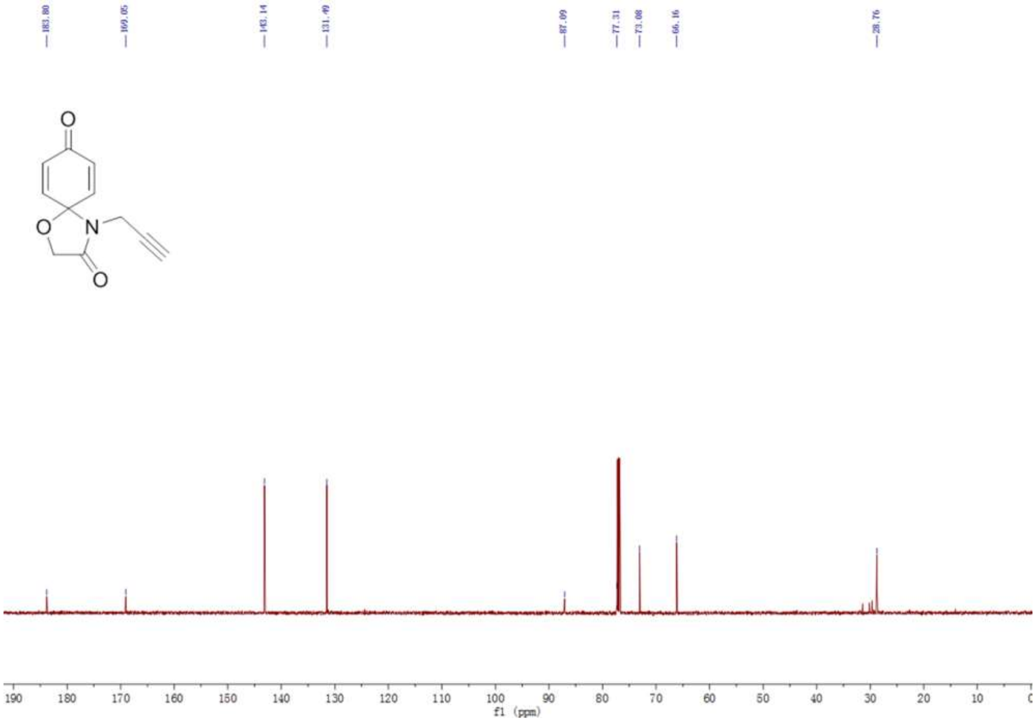

^{13}C NMR spectra of compounds **11c**

Compound **11d**

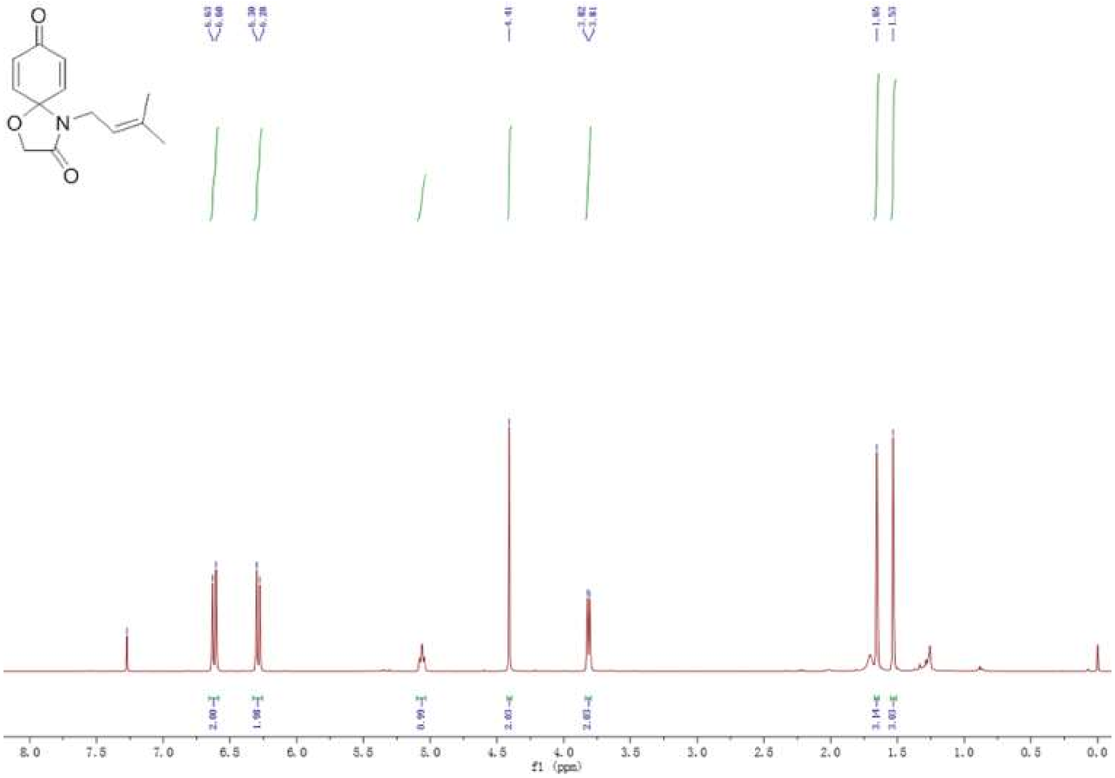

^{1}H NMR spectra of compound **11d**

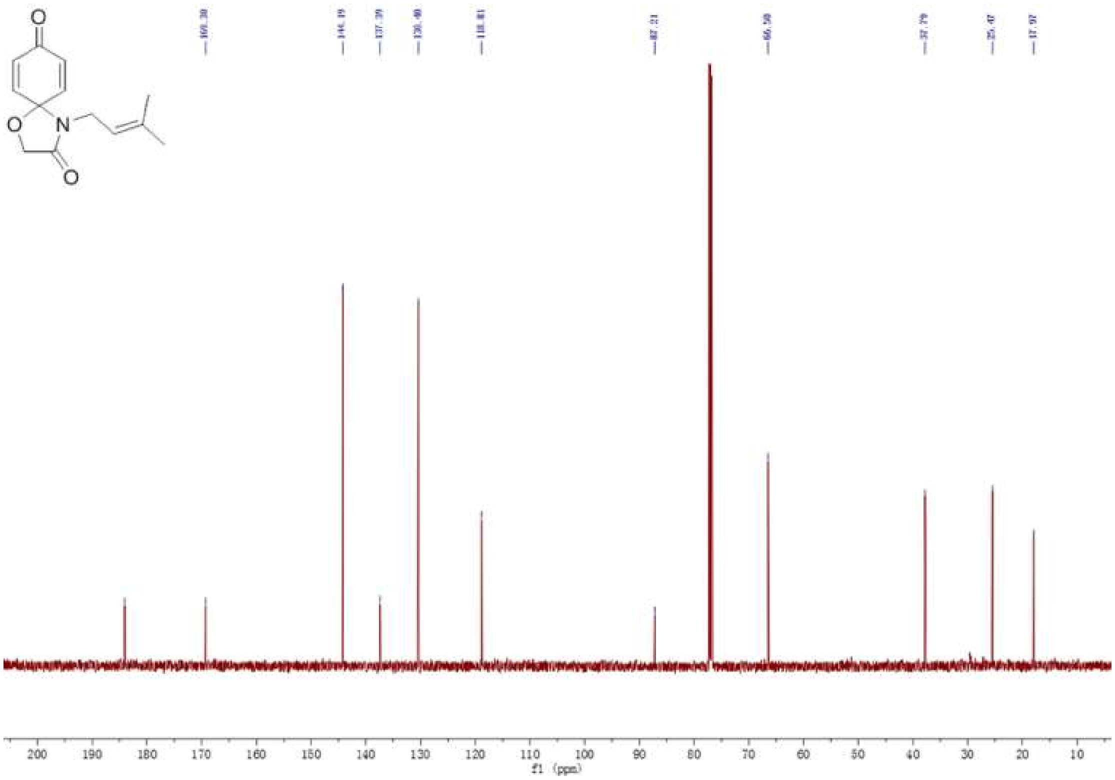

^{13}C NMR spectra of compounds **11d**

Compound **11e**

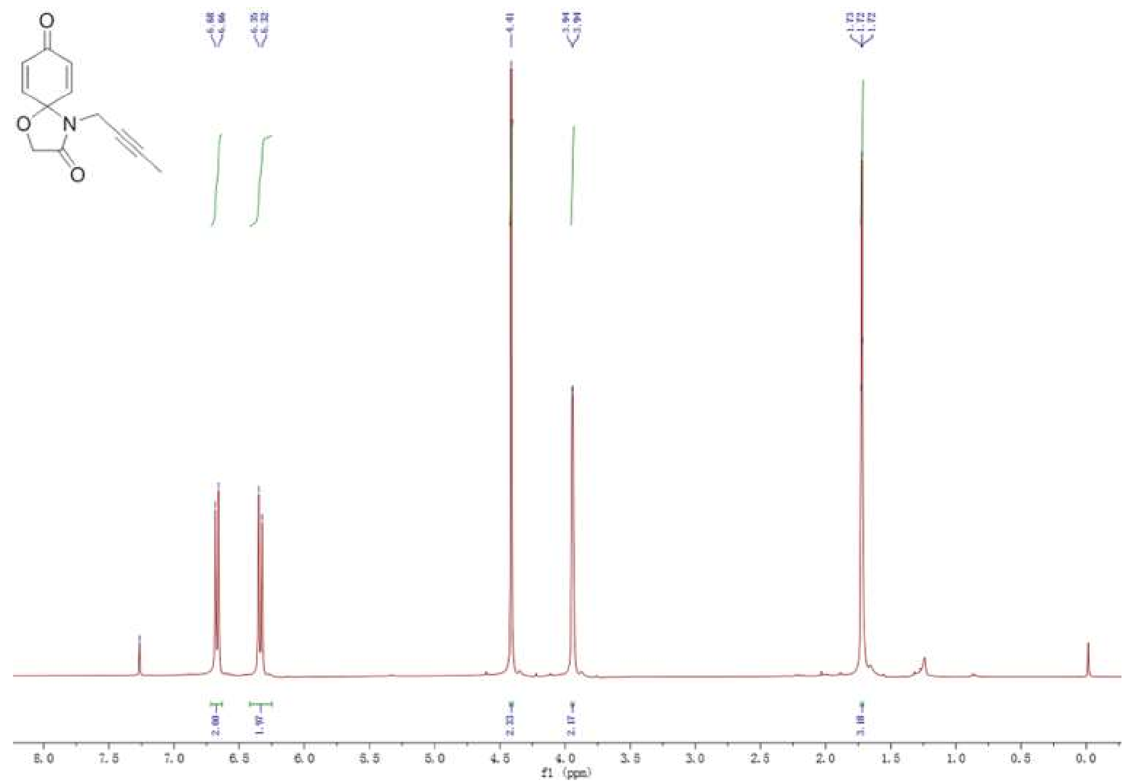

^1H NMR spectra of compound **11e**

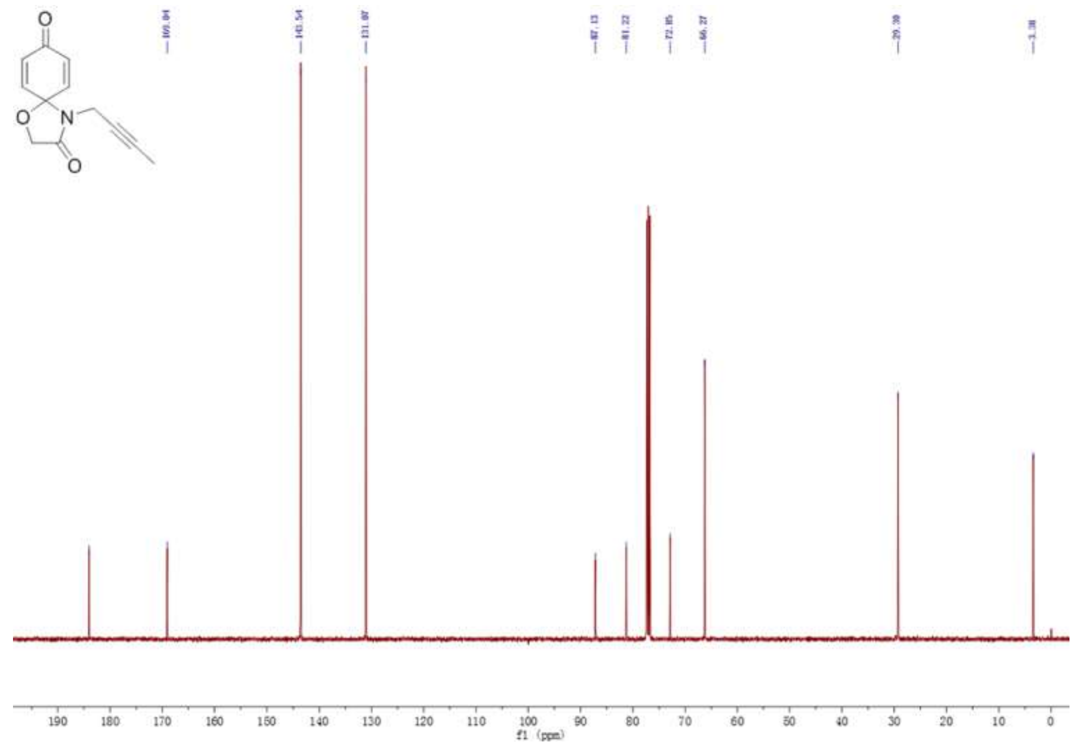

^{13}C NMR spectra of compounds **11e**

Compound **11f**

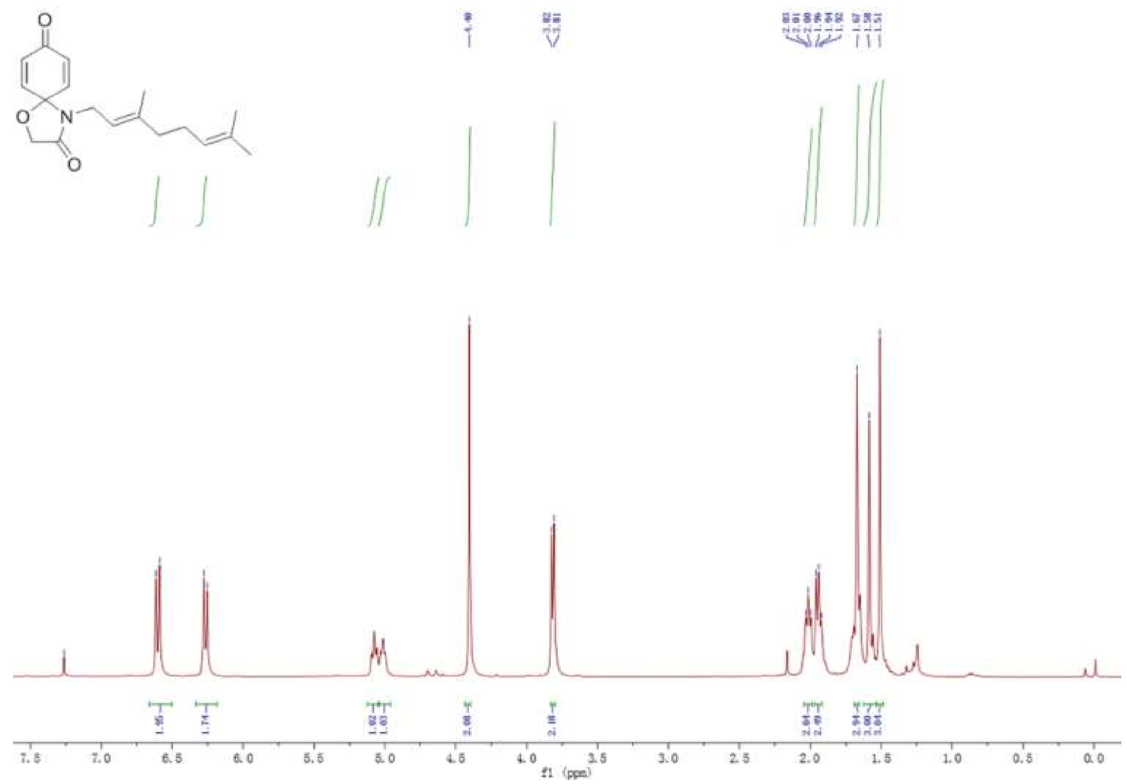

^{1}H NMR spectra of compound **11f**

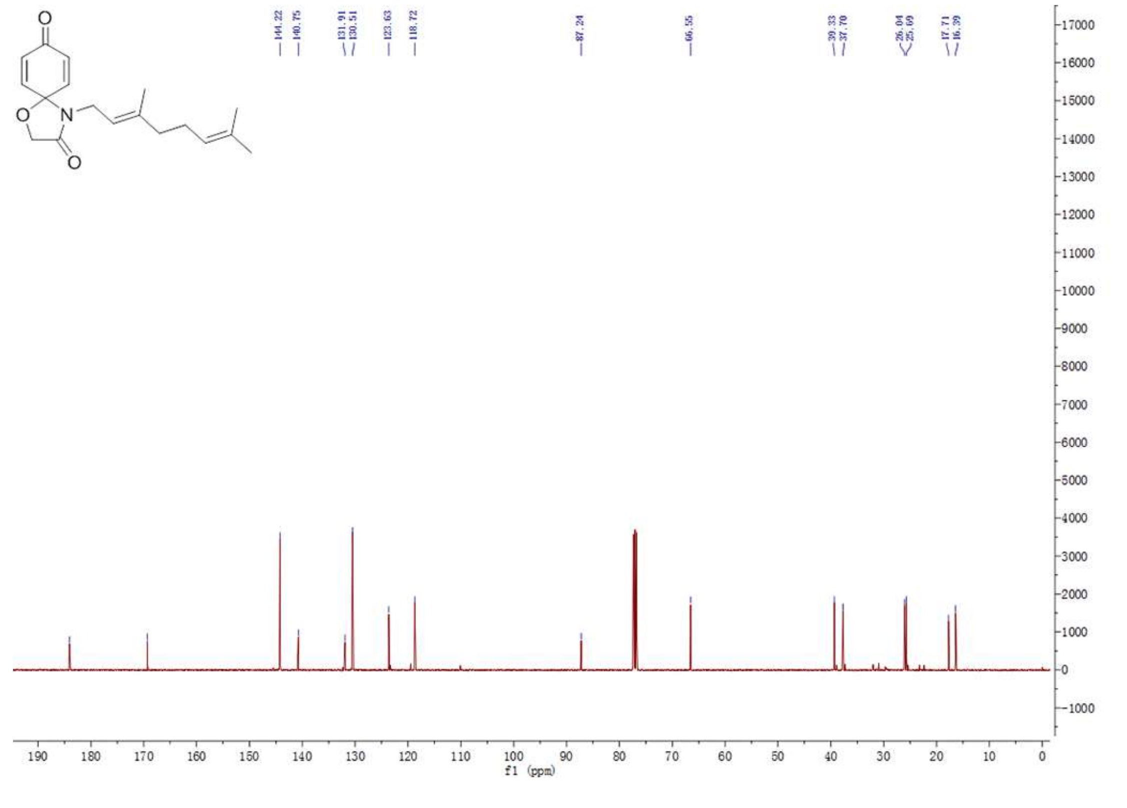

^{13}C NMR spectra of compounds **11f**

Compound **11g**

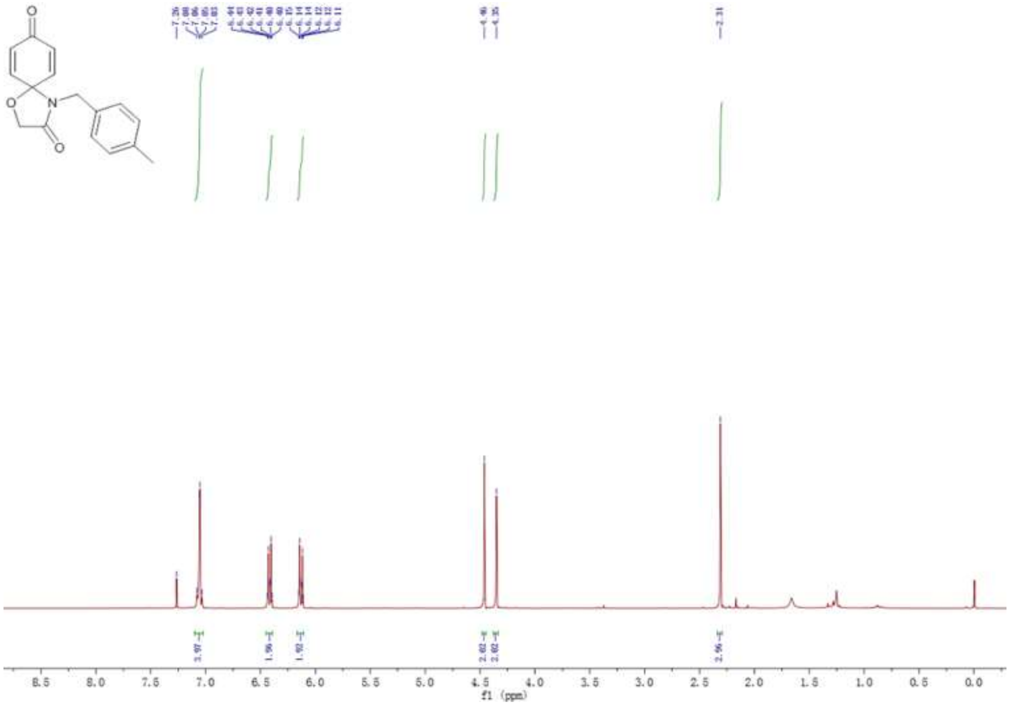

^{1}H NMR spectra of compound **11g**

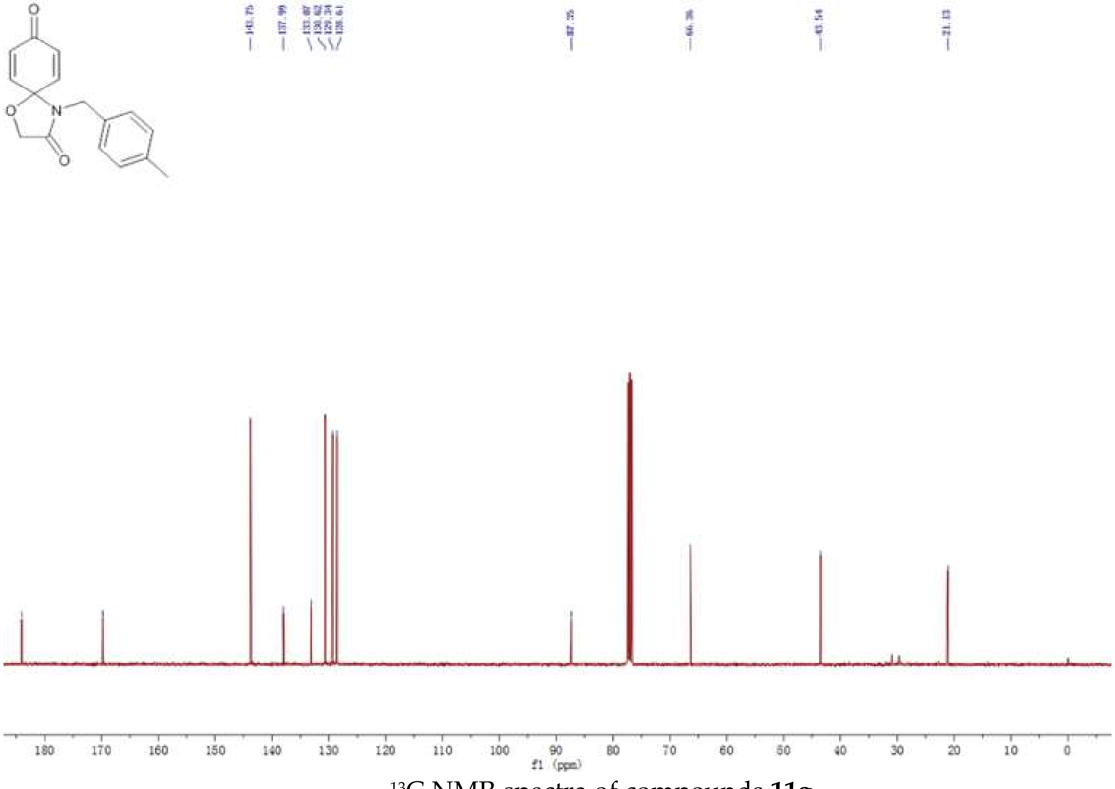

^{13}C NMR spectra of compounds **11g**

Compound **11h**

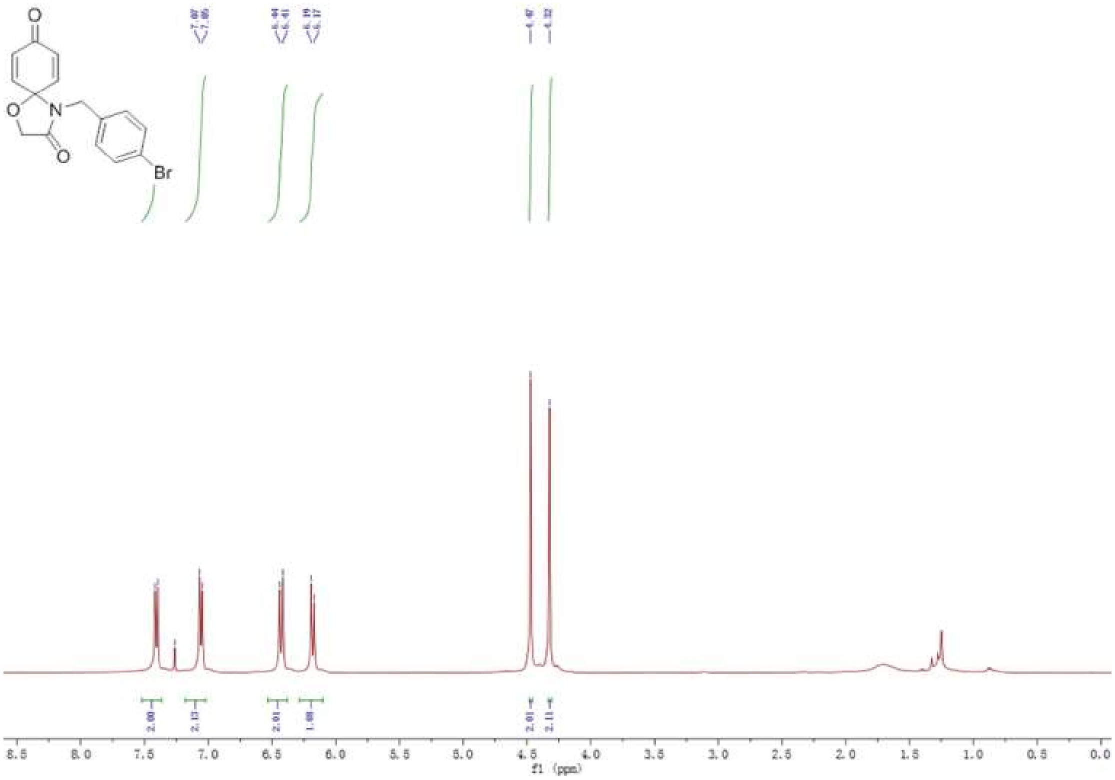

^{1}H NMR spectra of compound **11h**

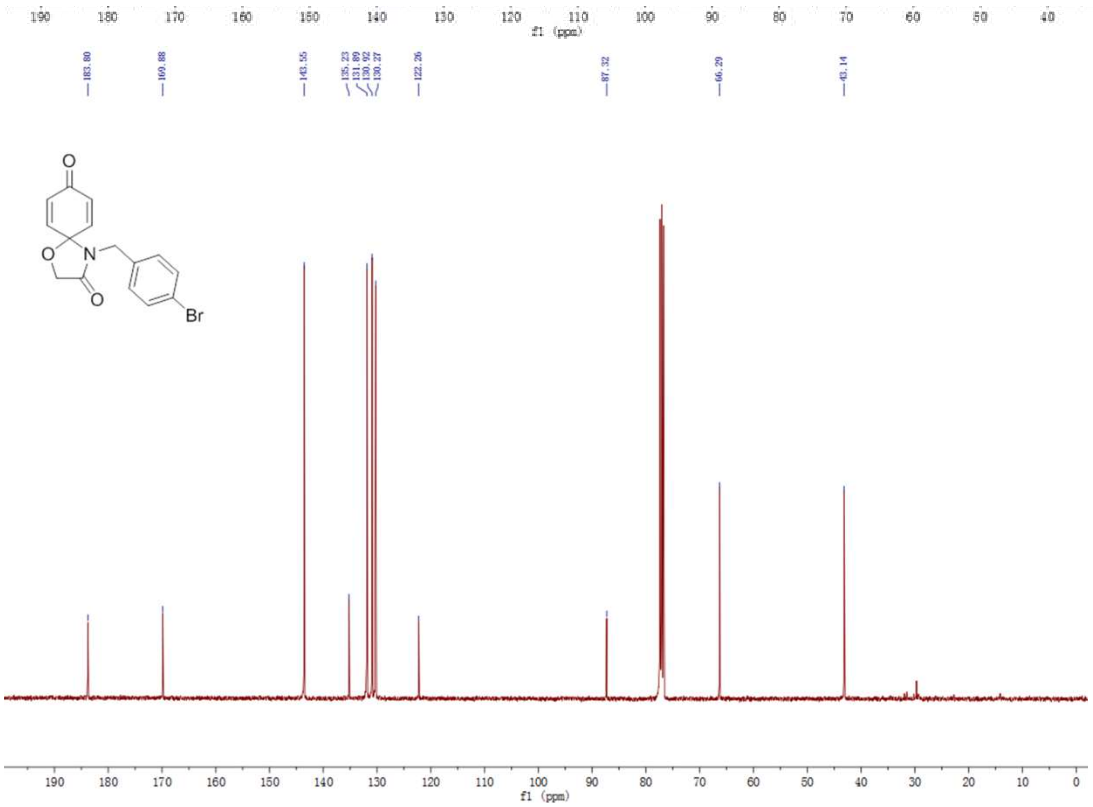

^{13}C NMR spectra of compounds **11h**

Compound **11i**

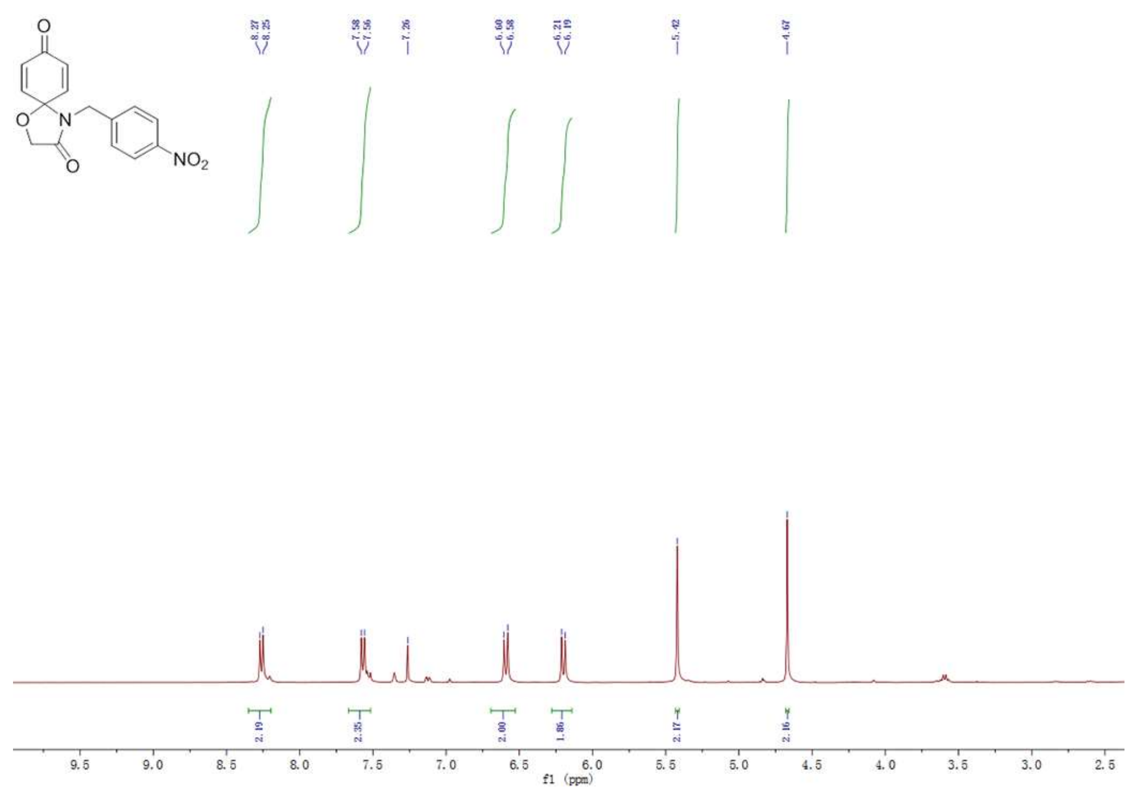

^{1}H NMR spectra of compound **11i**

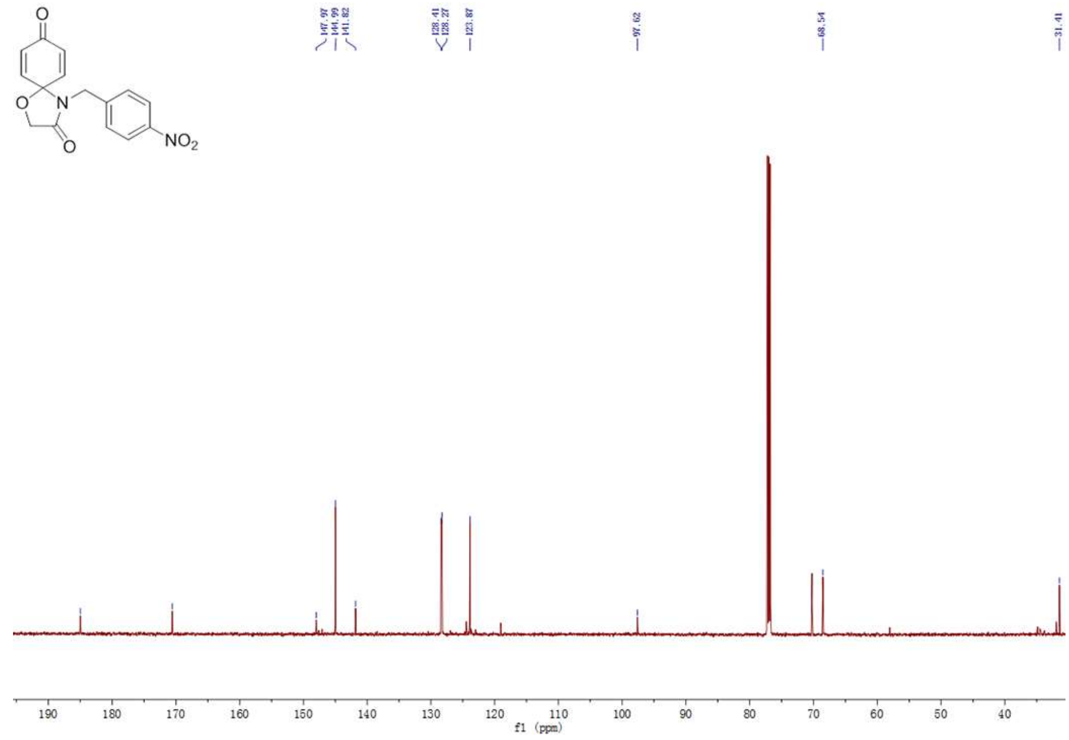

^{13}C NMR spectra of compounds **11i**

Compound **11j**

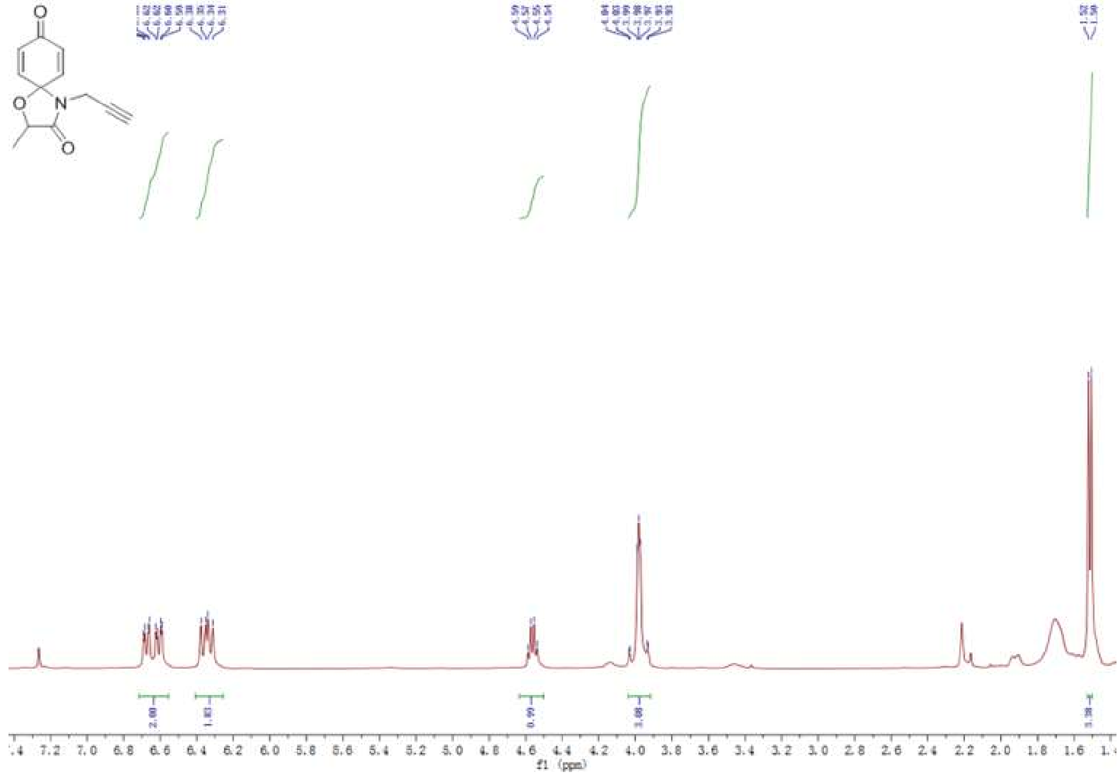

^{1}H NMR spectra of compound **11j**

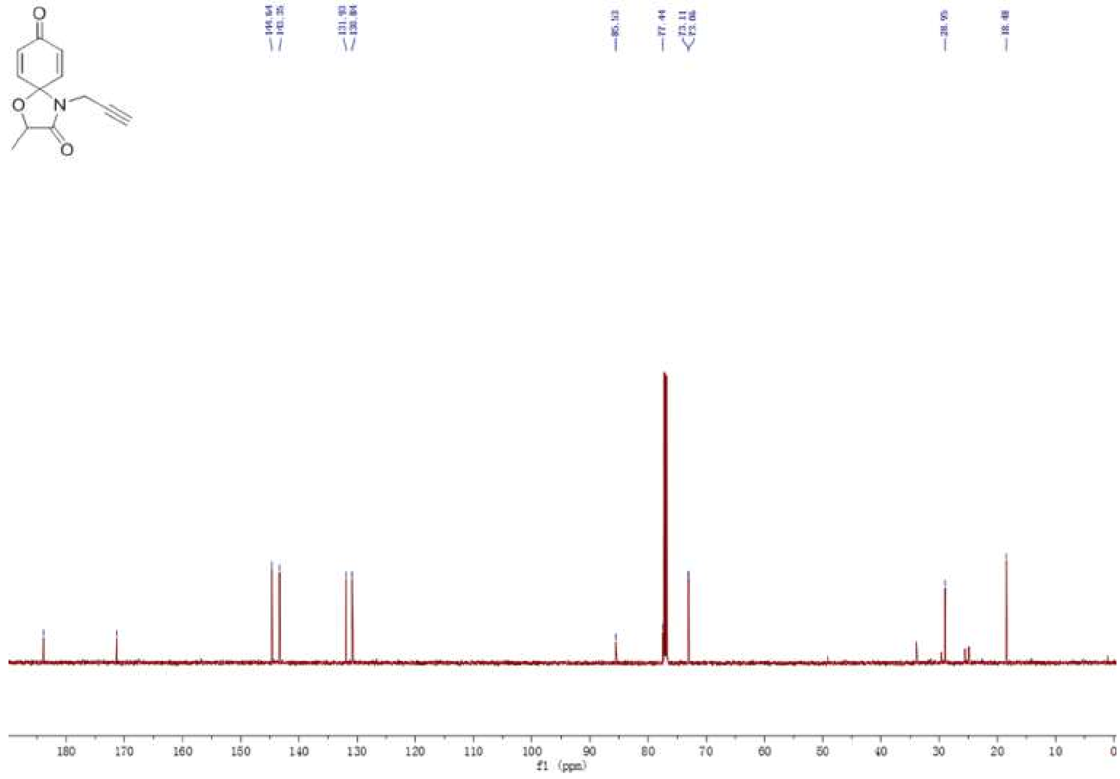

^{13}C NMR spectra of compounds **11j**

Compound **11k**

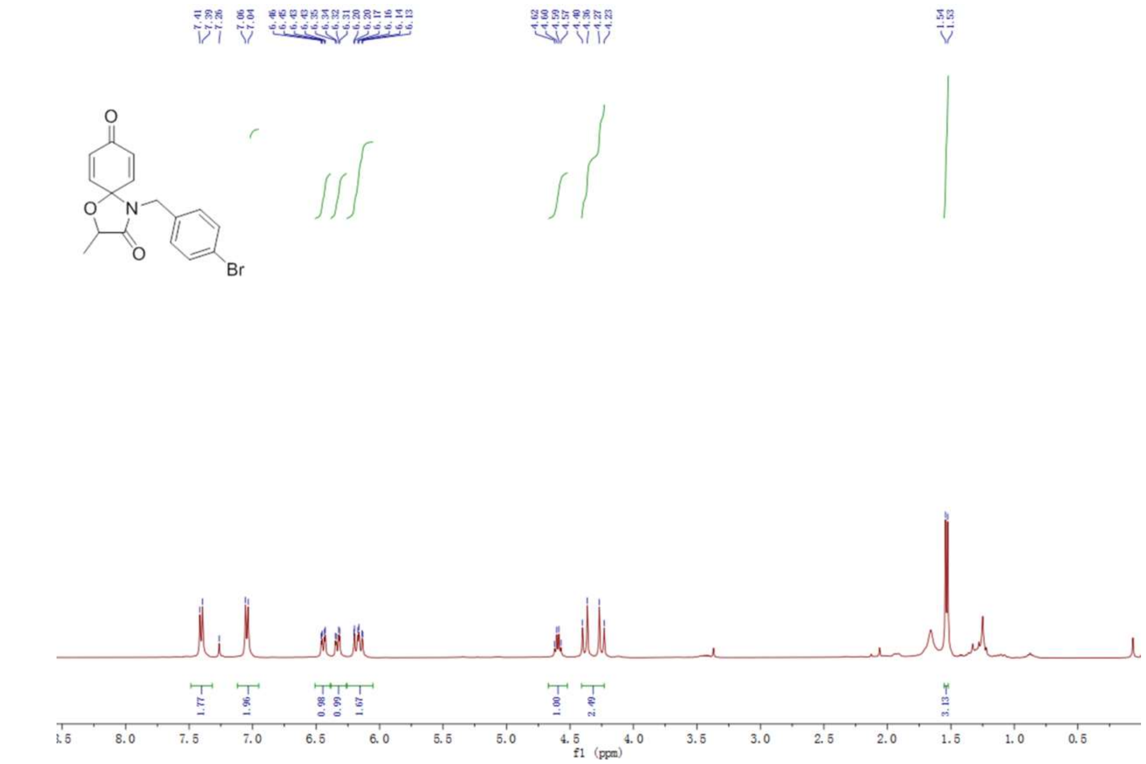

^{1}H NMR spectra of compound **11k**

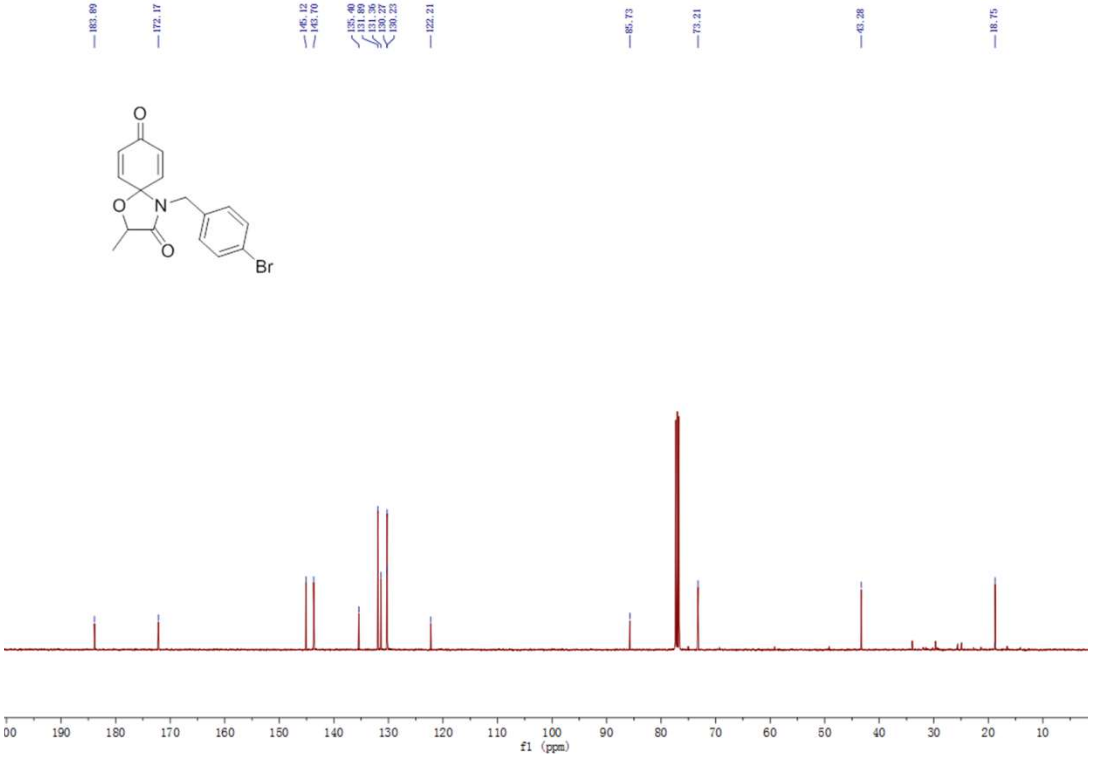

^{13}C NMR spectra of compounds **11k**

Compound **12a**

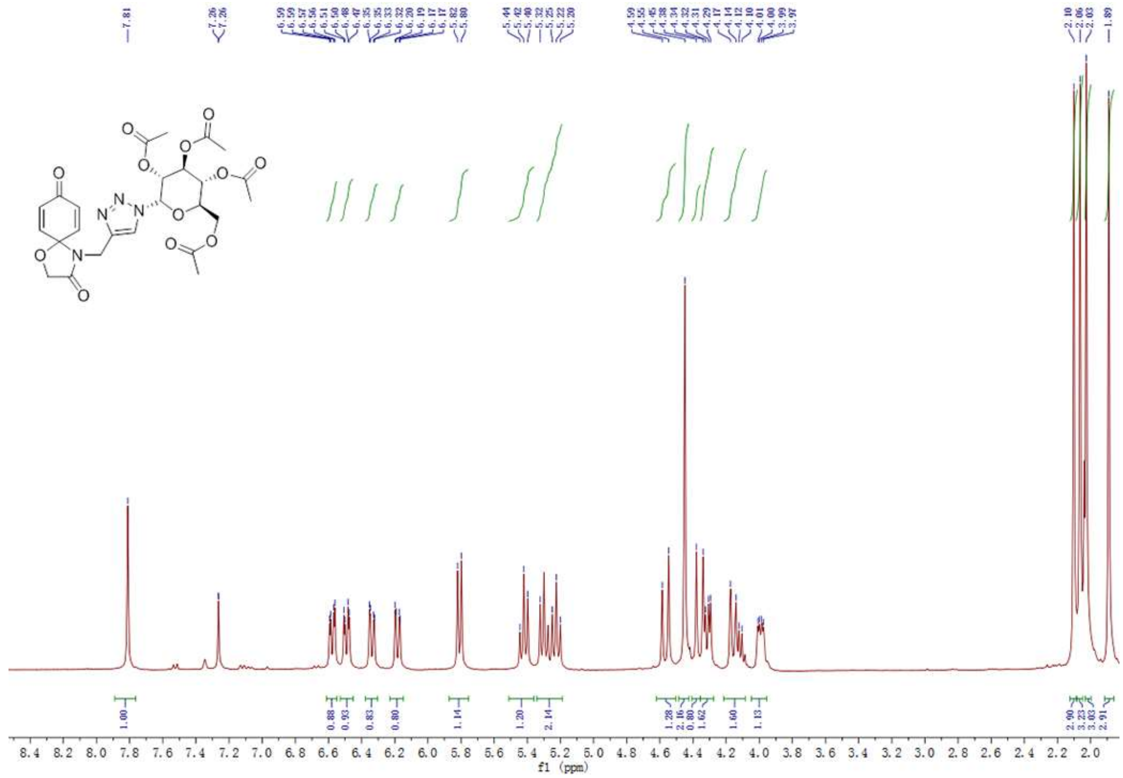

^{1}H NMR spectra of compound **12a**

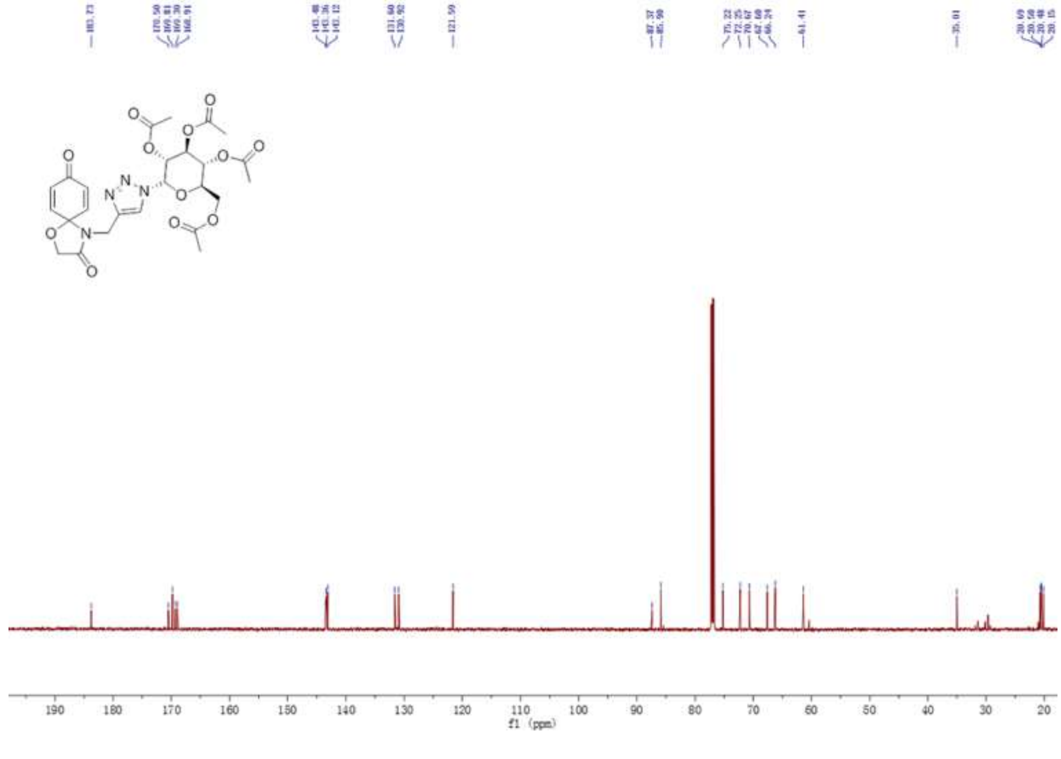

^{13}C NMR spectra of compounds **12a**

Compound **12b**

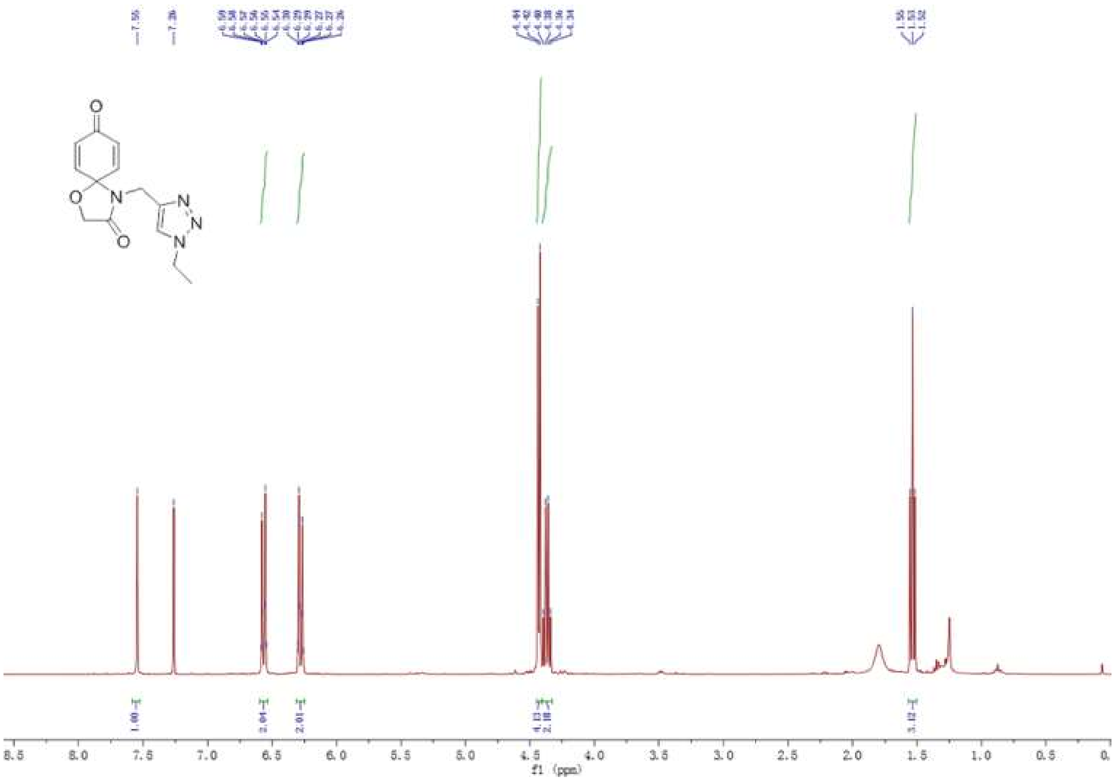

^{1}H NMR spectra of compound **12b**

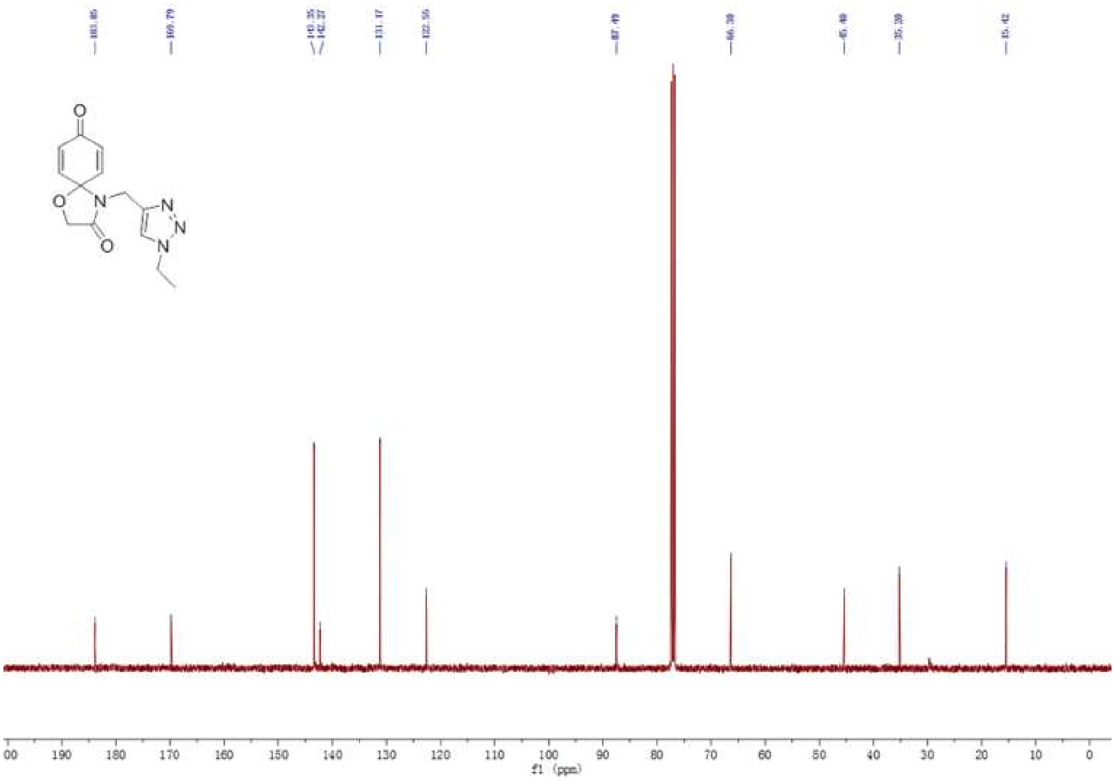

^{13}C NMR spectra of compounds **12b**

Compound **12c**

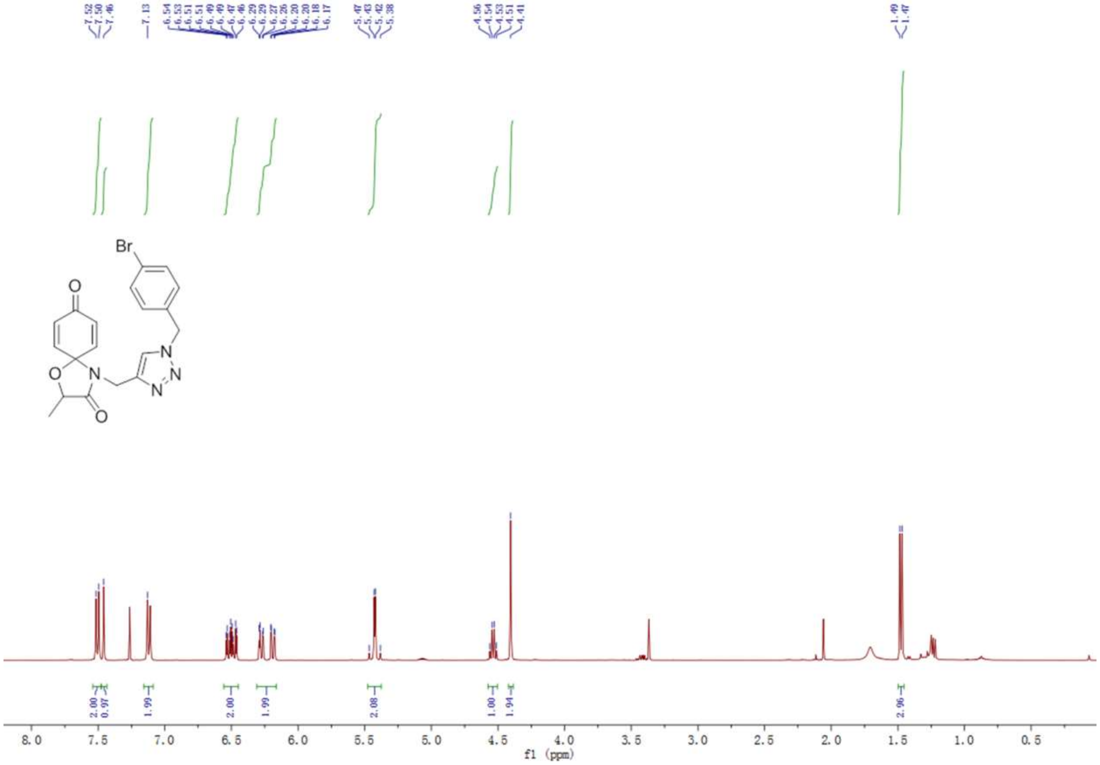

^{1}H NMR spectra of compound **12c**

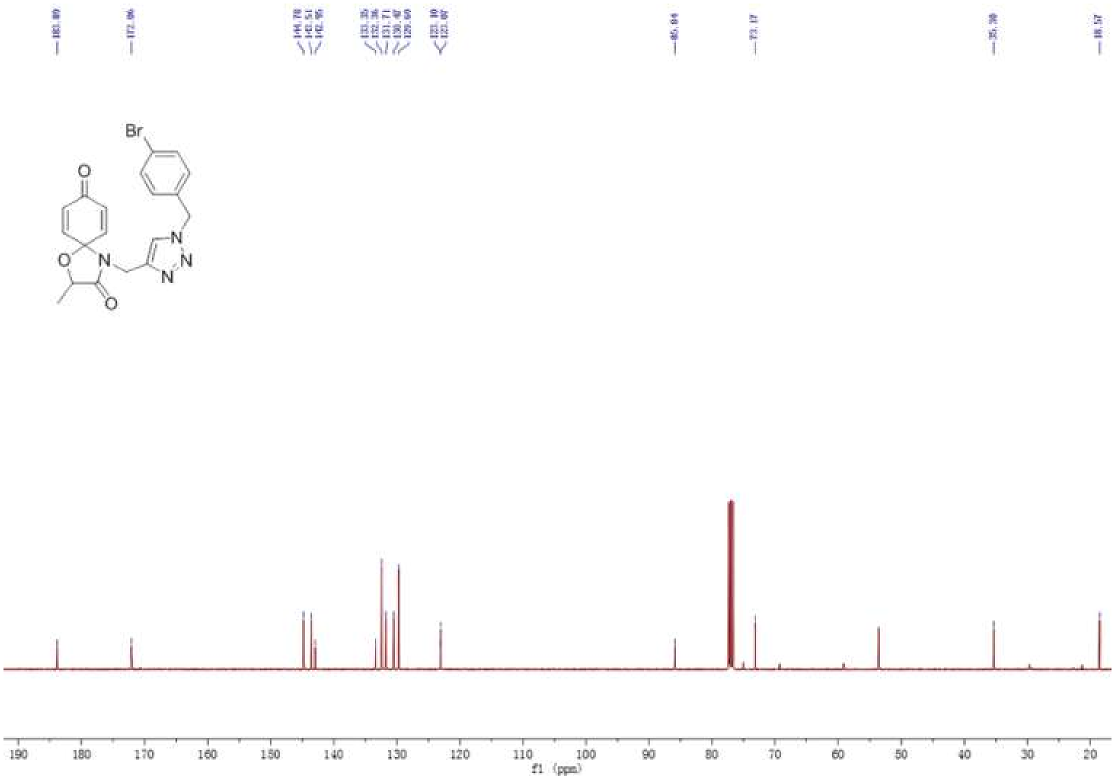

^{13}C NMR spectra of compounds **12c**

Compound **12d**

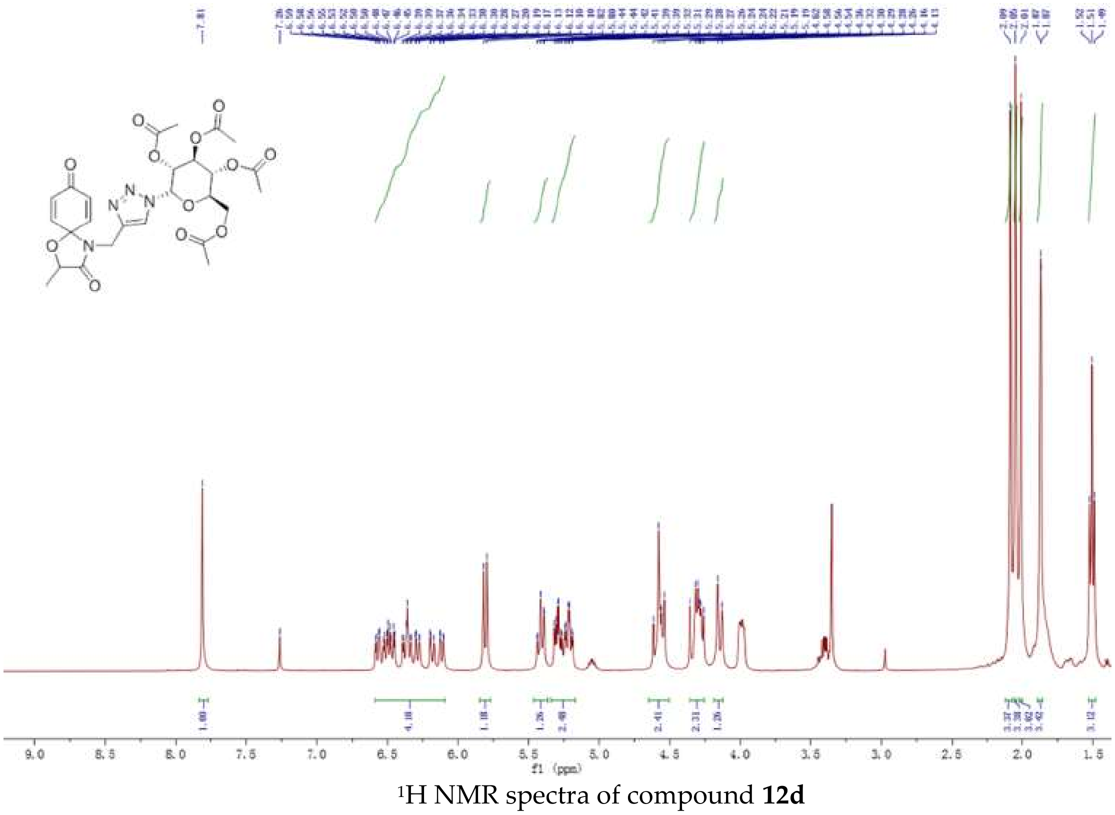

^{1}H NMR spectra of compound **12d**

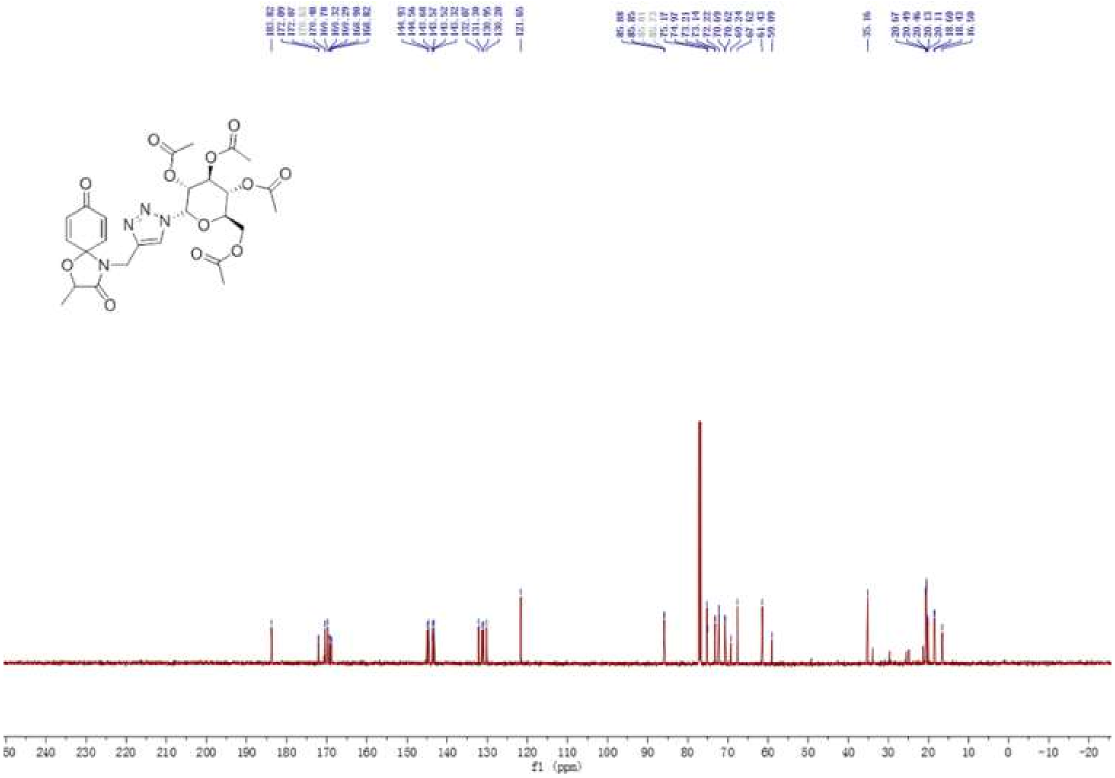

^{13}C NMR spectra of compounds **12d**
